# Metabolic perturbation of *Streptomyces albulus* by introducing NADP-dependent glyceraldehyde 3-phosphate dehydrogenase

**DOI:** 10.3389/fmicb.2024.1328321

**Published:** 2024-01-24

**Authors:** Jiaqi Mao, Min Zhang, Wenjuan Dai, Chenghao Fu, Zhanzhan Wang, Xiuwen Wang, Qingshou Yao, Linghui Kong, Jiayang Qin

**Affiliations:** School of Pharmacy, Binzhou Medical University, Yantai, China

**Keywords:** *Streptomyces albulus*, metabolic perturbation, NADP-dependent glyceraldehyde 3-phosphate dehydrogenase, transcriptome and metabolome, ε-poly-L-lysine, bioactive natural products

## Abstract

The available resources of *Streptomyces* represent a valuable repository of bioactive natural products that warrant exploration. *Streptomyces albulus* is primarily utilized in the industrial synthesis of ε-poly-L-lysine (ε-PL). In this study, the NADP-dependent glyceraldehyde 3-phosphate dehydrogenase (GapN) from *Streptococcus mutans* was heterologously expressed in *S. albulus* CICC11022, leading to elevated intracellular NADPH levels and reduced NADH and ATP concentrations. The resulting perturbation of *S. albulus* metabolism was comprehensively analyzed using transcriptomic and metabolomic methodologies. A decrease in production of ε-PL was observed. The expression of *gapN* significantly impacted on 23 gene clusters responsible for the biosynthesis of secondary metabolites. A comprehensive analysis revealed a total of 21 metabolites exhibiting elevated levels both intracellularly and extracellularly in the *gapN* expressing strain compared to those in the control strain. These findings underscore the potential of *S. albulus* to generate diverse bioactive natural products, thus offering valuable insights for the utilization of known *Streptomyces* resources through genetic manipulation.

## 1 Introduction

Natural products (NPs) generated from microorganisms and their derivatives are important sources of bioactive substances such as antibiotics, pesticides, antiparasites, anti-infectives, and antitumor drugs (Al-shaibani et al., 2021; Jose et al., 2021; Reddy et al., 2021). Among all the microorganisms, Actinomycetes contribute significantly to the production of bioactive compounds, including well-known secondary metabolites (SMs), such as streptomycin, vancomycin, erythromycin, rifamycin and tetracycline (Kong et al., 2019; Al-shaibani et al., 2021; Jose et al., 2021; Reddy et al., 2021). To obtain new bioactive compounds, scientists have strived to screen Actinomycetes, especially *Streptomyces*, from a variety of environments, including marine and extreme environments (Donald et al., 2022). Another resource that should not be overlooked is the known *Streptomyces*. The number of *Streptomyces* genomes that have been sequenced is continuously increasing, and a large number of secondary metabolite biosynthesis gene clusters (SM-BGCs) have been discovered (Belknap et al., 2020; Lee et al., 2020). However, the majority of these BGCs, are silent when tested under standard laboratory conditions (Liu et al., 2021). Therefore, numerous strategies, including heterologous expression (Kang and Kim, 2021), overexpression in native host (Li H. et al., 2022), promoter engineering (Liu X. F. et al., 2022), transcriptional regulation engineering (Li et al., 2019; Martínez-Burgo et al., 2019) and ribosome engineering (Zhang et al., 2019; Li et al., 2021) have been developed to activate these BGCs in NP discovery studies (Beck et al., 2021; Liu et al., 2021). However, since most of these studies focused on one or more metabolites, the full potential of *Streptomyces* has rarely been studied.

*Streptomyces albulus* is a species of *Streptomyces* whose main SM is ε-poly-L-lysine (ε-PL); thus, it has become an industrial production strain for ε-PL (Wang et al., 2021; Li S. et al., 2022). ε-PL is an L-lysine homopolymer with broad-spectrum antibacterial activity and thus has a wide range of uses in the food, medical, chemical, and other industries (Wang et al., 2021). *S. albulus* is also capable of synthesizing other bioactive substances, such as wuyiencin (Liu B. et al., 2022; Yang et al., 2022), salinomycin (Zhang et al., 2019), tetramycin A and B (Yamanaka et al., 2020), and toyocamycin (Liu B. et al., 2022). In our previous study, we sequenced the genome of *S. albulus* CICC11022 and subsequently predicted 37 SM-BGCs on its chromosome (Lian et al., 2022). These results indicate that the metabolism of *S. albulus* CICC11022 is complicated, and the potential of this strain to produce bioactive compounds needs to be further clarified.

Glyceraldehyde 3-phosphate dehydrogenase (GAPDH) is a glycolytic enzyme that catalyze the conversion of glyceraldehyde 3-phosphate into 1,3-bisphosphoglycerate. This reaction is usually accompanied by the reduction of NAD^+^ to produce NADH. However, another type of GAPDH, GapN (EC 1.2.1.9), is found in nature. GapN is found in both photosynthetic organisms (Mateos and Serrano, 1992) and some Gram-positive bacteria, such as *Streptococcus mutans* (Boyd et al., 1995). GapN catalyzes the non-reversible oxidation of glyceraldehyde 3-phosphate to 3-phosphoglycerate along with NADPH formation. Since four molecules of NADPH are required for the biosynthesis of one molecule of L-lysine in *Corynebacterium glutamicum*, the expression of *gapN* can alleviate the dependence of cells on the pentose phosphate pathway and thus increase the production of L-lysine (Bommareddy et al., 2014; Takeno et al., 2016; Wu et al., 2019).

The biosynthesis of ε-PL in *S. albulus* involves the diaminopimelate pathway, as in *C. glutamicum* and uses L-lysine as a precursor (Lian et al., 2022). The impact of *gapN* expression on ε-PL production in *S. albulus* has yet to be determined, despite its favorable effect on L-lysine production in *C. glutamicum*. In the present study, *gapN* from *Streptococcus mutans* was heterologously expressed in *S. albulus* CICC11022. The effects of *gapN* expression on the metabolism of *S. albulus* CICC11022 were determined using transcriptomic and metabolomic approaches. The potential of *S. albulus* CICC11022 to synthesize various NPs was subsequently emphasized.

## 2 Materials and methods

### 2.1 Strains, plasmids, and culture conditions

The strains, plasmids and primers used for strain construction are listed in Supplementary Table 1. The host bacterium and vector used to express *gapN* were *S. albulus* CICC11022 and pSET152, respectively (Wang et al., 2020). Spores of *S. albulus* strains were cultured on MS solid medium containing 20 g/L mannitol, 20 g/L soybean powder, and 20 g/L agar powder. The seed culture was prepared using M3G medium composed of 50 g/L glucose, 10 g/L (NH_4_)_2_SO_4_, 5 g/L yeast extract, 0.5 g/L MgSO_4_⋅7H_2_O, 0.8 g/L K_2_HPO_4_, 1.36 g/L KH_2_PO_4_, 0.03 g/L FeSO_4_⋅7H_2_O, and 0.04 g/L ZnSO_4_⋅7H_2_O at an initial pH of 6.8. The fermentation medium for *S. albulus* was the M3G medium supplemented with 5 g/L sodium citrate. Cell growth and SM production ability were compared by initially culturing *S. albulus* strains on the MS solid medium for 5–6 days at 30°C. Afterward, the spores were collected, inoculated into 50 mL of M3G medium, and cultured at 30°C and 220 rpm for 48 h. Finally, 5 mL of the seed culture was inoculated into 50 mL of the fermentation medium and cultured at 30°C and 220 rpm for 168 h. Cells of *S. albulus* Q-152 and Q-gapN were harvested after 48 h of fermentation for RNA sequencing, as was the quantification of NAD(P)H and ATP. Samples for metabolomics analysis were collected after 7 days of fermentation. *E. coli* strains were aerobically cultured at 37°C in Luria–Bertani (LB) medium, which contained 10 g/L tryptone, 5 g/L yeast extract, and 10 g/L sodium chloride. When needed, antibiotics were used at the following concentrations: 50–80 μg/mL apramycin, 25–50 μg/mL chloramphenicol, 40–50 μg/mL kanamycin, and 25 μg/mL nalidixic acid.

### 2.2 Construction of the *gapN* heterologous expression strain

The SP43 promoter, the SR41 ribosome-binding site (RBS) (Bai et al., 2015), and the codon-optimized *gapN* gene (accession number: OR257568) from *Streptococcus mutans* GS-5 were chemically synthesized (Supplementary Figure 1). The primers gapN-F and gapN-R were subsequently used to amplify the SP43-SR40-gapN DNA fragment. SP43-SR40-gapN was digested using *Xba*I and *Bam*HI, ligated to pSET152 and subsequently transformed into *E. coli* Trans5α. After screening and verification, the recombinant expression plasmid was named pSET152-gapN, which was subsequently transferred into *E. coli* ET12567/pUZ8002 and then into *S. albulus* CICC11022 via intergeneric conjugation in accordance with a previously reported method (Lian et al., 2022). The obtained *gapN*-expressing strain, *S. albulus* Q-gapN, harbored pSET152-gapN at the *attB* site of its chromosome. Moreover, a control strain, *S. albulus* Q-152, which harbors pSET152, was also constructed.

### 2.3 RNA sequencing and transcriptomic analysis

The total RNA of *S. albulus* Q-152 (control) and Q-gapN (gapN) was isolated after 48 h of fermentation. Three biological replicates were prepared for each group. An Illumina HiSeq 2000 platform (Majorbio Bio-Pharm Technology Co., Ltd., Shanghai, China) was used for RNA sequencing. The sequencing data were deposited in the NCBI Sequence Read Archive under accession numbers SRR20727629 (control) and SRR20727628 (gapN). The online platform Majorbio Cloud Platform^1^ was used for conducting Gene Ontology (GO) and Kyoto Encyclopedia of Genes and Genomes (KEGG) analyses, facilitating functional annotation, classification, and enrichment analysis of the genes (Ren et al., 2022). Additionally, the genome sequence of *S. albulus* CICC11022 (BioProject accession number: PRJNA859656) served as a reference. Genes that exhibited twofold or greater changes [fold change (FC) ≥ 2 or ≤ -2; FDR < 0.05] between samples were defined as differentially expressed genes (DEGs). Functional enrichment analyses were performed as previously reported (Lian et al., 2022).

### 2.4 qRT-PCR

The *gapN* gene expression levels in *S. albulus* Q-gapN and Q-152 were compared via quantitative real-time PCR (qRT-PCR) using the primers RT-gapN-F and RT-gapN-R (Supplementary Table 3). qRT-PCR was also used to verify the RNA sequencing data. The genes and primers used are shown in Supplementary Table 2. All samples were taken after 48 h of fermentation. The PCR experiments were performed as reported previously (Lian et al., 2022).

### 2.5 Untargeted metabolomic analysis

The effect of *gapN* expression on the intracellular and extracellular metabolites of *S. albulus* was investigated using an untargeted metabolomic method. The samples were divided into four groups, representing two strains. The intracellular and extracellular metabolite samples of the *gapN*-expressing strain were labeled gapN and gapN_s, while the intracellular and extracellular metabolite samples of the control strain Q-152 were labeled control and control_s, respectively. Cells and cell culture supernatants were taken after 7 days of fermentation. Six biological replicates were prepared for each group. Liquid chromatography-mass spectrometry (LC-MS)-based metabolomic detection was conducted following a previously reported methodology (Lian et al., 2022). The LC-MS sample preparation procedure was conducted as follows: A precisely weighed 50 mg of cell or supernatant sample was placed into a 2 mL centrifuge tube, followed by the addition of a ground bead with a diameter of 6 mm and 400 μL of methanol and acetonitrile in equal proportions. Subsequently, the samples were subjected to grinding using a frozen tissue grinder for 6 min at −10°C and 50 Hz, followed by ultrasonic extraction for 30 min at 5°C and 40 kHz. Afterward, the samples were frozen at −20°C for 30 min and subsequently centrifuged at 13,000 × *g* and 4°C for 15 min. The next step involved redissolving the samples by adding 120 μL of solution composed of acetonitrile and water in equal proportions. The mixture was then vortexed for 30 s and subjected to an additional ultrasonic extraction for five more minutes. Following another round of centrifugation, the resulting supernatant was collected and utilized in LC-MS analysis. The UHPLC-Q Exactive HF-X system, equipped with a Q-Exactive quadrupole-Orbitrap mass spectrometer and a heated electrospray ionization (ESI) source (Thermo Fisher Scientific, Waltham, MA, USA), was employed as the analytical instrument for metabolic profiling analysis in both ESI-positive and ESI-negative ion modes. The column used was ACQUITY UPLC HSS T3 (100 mm × 2.1 mm i.d., 1.8 μm; Waters, Milford, USA). Mobile phase A consisted of 95% water and 5% acetonitrile (with 0.1% formic acid), while mobile phase B consisted of 47.5% acetonitrile, 47.5% isopropanol, and 5% water (containing 0.1% formic acid). The injection volume was 3 μL, and the column temperature was 40°C. Quality control samples were prepared by combining equal volumes of extracts from all samples. LC-MS raw data were acquired and processed using ProgenesisQI software (Waters Corporation, Milford, USA). Subsequently, the software was used to search for characteristic peaks and identify metabolites by matching MS and tandem mass spectra (MS/MS) information with the metabolic databases under an MS mass error threshold of less than 10 ppm. The databases utilized included the HMDB,^2^ the Metlin database,^3^ and a proprietary database developed by Majorbio Bio-Pharm Technology Co., Ltd., Shanghai, China. Differentially abundant metabolites with variable importance plot (VIP) values > 1 and *P* ≤ 0.05 were screened.

### 2.6 Analytical method

NADPH and NADH concentrations were measured using an NADP^+^/NADPH Assay Kit (Beyotime, China) and an NAD^+^/NADH Assay Kit (Beyotime, China), respectively, based on the WST-8 method (Chamchoy et al., 2019). The ATP concentration was determined using a firefly luciferase-based ATP Assay Kit (Beyotime, China) (Lundin, 2000). An Enhanced BCA Protein Assay Kit (Beyotime, China) was used to determine the protein concentrations of the cells used for NADPH, NADH, and ATP determination. The NADPH, NADH, and ATP concentrations per microgram of protein were subsequently calculated.

Cell growth and glucose concentration were measured by using a spectrophotometer and an SBA-40E biosensor analyzer, respectively (Wang et al., 2020). The concentration of ε-PL was measured as described previously (Lian et al., 2022). The concentrations of anisomycin were determined using a previously described method (Shen et al., 2019). GraphPad Prism 8.3 (GraphPad Software, USA) was used to plot the results and for statistical analysis.

## 3 Results

### 3.1 Effects of *gapN* expression on intracellular NADPH, NADH, and ATP levels

Initially, a heterologous *gapN* gene expression strain was constructed. A schematic diagram of the expression vector pSET152-gapN is shown in Supplementary Figure 2A. The *gapN* expression levels in *S. albulus* Q-gapN and Q-152 were compared via qRT-PCR (Supplementary Figure 2B). These results indicate that the *gapN* gene heterologously expressed strain was successfully constructed. It was subsequently used in cofactor concentration detection, fermentation product analysis, and omics investigations.

**FIGURE 1:**
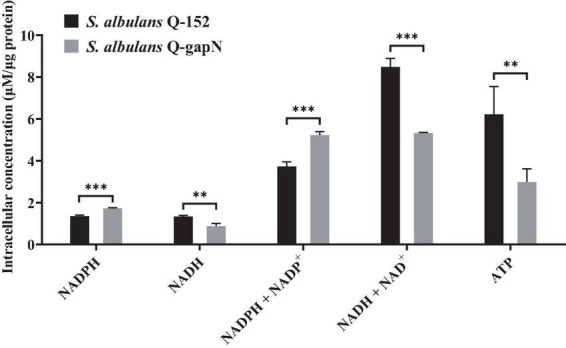
Effect of *gapN* expression on intracellular NADPH, NADH and ATP concentrations. **P < 0.01; ***P < 0.001.

The intracellular NADPH, NADH and ATP concentrations were subsequently determined to study the effect of heterologous *gapN* expression. As shown in Figure 1, the NADPH concentration in *S. albulus* Q-gapN was 1.74 ± 0.02 μM/μg protein, which was 28.0% greater than the 1.35 ± 0.05 μM/μg protein concentration in the control strain. Moreover, the NADH concentration in *S. albulus* Q-gapN was only 0.88 ± 0.13 μM/μg protein, which was 24.0% lower than the 1.34 ± 0.05 μM/μg protein concentration in the control strain. The total NADP^+^ (NADP^+^ + NAPDH) concentrations in *S. albulus* Q-gapN and Q-152 were 5.22 ± 0.16 and 3.73 ± 0.22 μM/μg protein, respectively. Moreover, the total NAD^+^ (NAD^+^ + NADH) concentrations in *S. albulus* Q-gapN and Q-152 were 5.36 ± 0.03 and 8.48 ± 0.41 μM/μg protein, respectively. These results suggest that the heterologous expression of *gapN* in *S. albulus* indeed perturbs the intracellular concentration balance between NADPH and NADH. We further examined the effect of this perturbation on the intracellular ATP content. The ATP concentration in *S. albulus* Q-gapN was 2.98 ± 0.63 μM/μg protein, which was 52.1% lower than the 6.22 ± 1.32 μM/μg protein concentration in the control strain (Figure 1).

### 3.2 Effects of *gapN* expression on cell growth and ε-PL production

**FIGURE 2:**
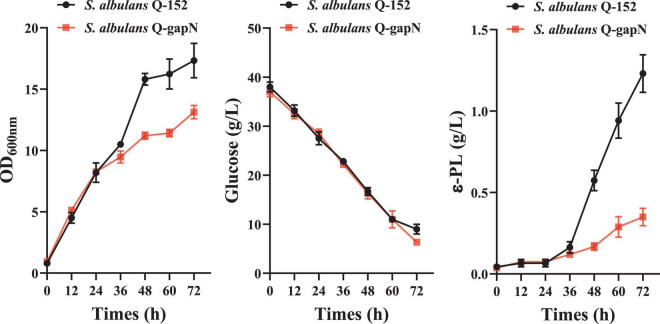
Effects of heterologous *gapN* expression on cell growth, glucose consumption, and ε-PL production.

The cell growth, residual glucose, and ε-PL production of *S. albulus* Q-gapN and Q-152 were subsequently compared; the results are shown in Figure 2. For the first 24 h, the cell growth and ε-PL production of both strains were similar. Afterward, significant differences were observed in the growth and production of the two strains. By 72 h, the maximum OD values of *S. albulus* Q-gapN and Q-152 were 13.13 and 17.33, respectively. The ε-PL concentrations produced by the Q-gapN and Q-152 strains were 0.35 and 1.23 g/L, respectively. Although the final ε-PL yields were quite different, the glucose consumption of the two strains was very similar. These results indicate that the heterologous expression of *gapN* resulted in slower strain growth and reduced ε-PL production.

### 3.3 Comparative transcriptomic analysis

**FIGURE 3:**
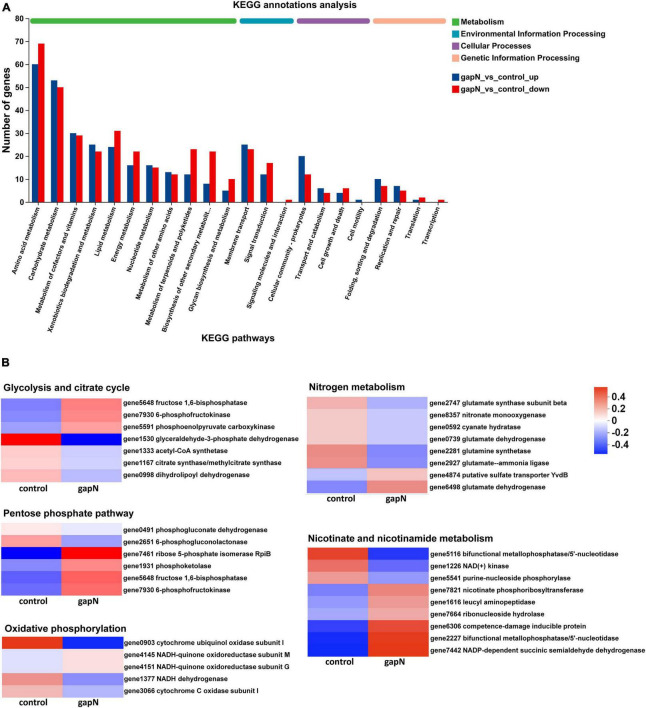
KEGG annotation analysis of the DEGs. **(A)** Classification of DEGs with KEGG annotation results. **(B)** Noteworthy DEGs within specific KEGG pathways.

A volcano plot of the significant DEGs between *S. albulus* Q-gapN (gapN) and Q-152 (control) is shown in Supplementary Figure 3A. The heterologous expression of *gapN* in *S. albulus* CICC11022 resulted in 2,935 DEGs, of which 1,318 and 1,617 genes were significantly up- and downregulated, respectively. Moreover, 76 and 208 sRNAs were found to be significantly up- and downregulated, respectively, by *gapN* expression. The accuracy of the transcriptome results was verified by qRT-PCR (Supplementary Table 3). The distribution of DEGs across various KEGG pathways (Ren et al., 2022) is presented in Figure 3A. Amino acid metabolism was the primary KEGG pathway associated with the highest number of DEGs, followed by carbohydrate metabolism. Noteworthy DEGs within the KEGG pathway are listed in Figure 3B, while additional information can be found in Supplementary Table 4. KEGG enrichment analyses of all the DEGs revealed that the most significantly regulated pathways in the *gapN* expressing strain were “steroid degradation” (KEGG: ko00984), “beta-alanine metabolism” (KEGG: ko00410), “beta-lactam resistance” (KEGG: ko01501), “C5-branched dibasic acid metabolism” (KEGG: ko00660), and “xylene degradation” (KEGG: ko00622) (*P* < 0.05) (Supplementary Figure 3B). GO enrichment analyses of all the DEGs revealed that the most significantly regulated GO terms in the *gapN* expressing strain were “iron ion binding” (GO:0005506), “monooxygenase activity” (GO:0004497), “heme binding” (GO:0020037), “tetrapyrrole binding” (GO:0046906), and “modified amino acid binding” (GO:0072341), among others (*FDR* < 0.001) (Supplementary Figure 3C).

### 3.4 Comparative metabolomic analysis

Liquid chromatography-mass spectrometry-based metabolomic approaches were used to study the effects of *gapN* expression on the intracellular and extracellular metabolites of *S. albulus*. A total of 10,494 positive ion peaks and 8,700 negative ion peaks were detected in all four groups. Partial least squares-discriminant analysis (PLS-DA) and Venn plot analysis demonstrated global metabolic changes in the four groups (Supplementary Figures 4A–D).

**FIGURE 4:**
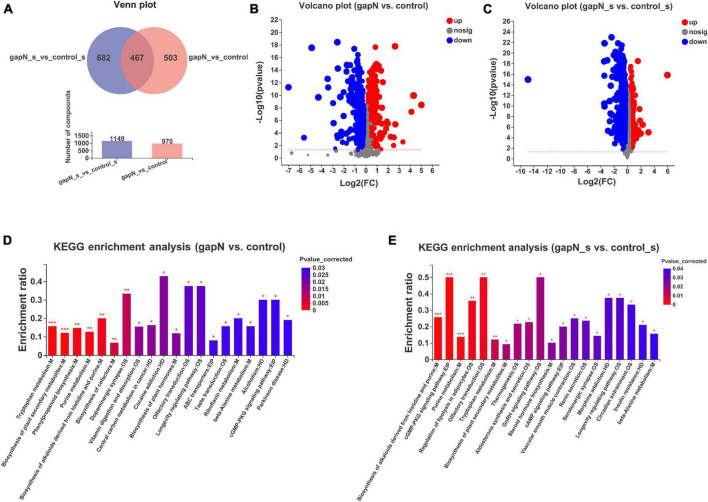
Intracellular and extracellular metabolomic comparisons of the *gapN*-expressing strain and the control strain. **(A)** Venn plot of the differential intracellular and extracellular metabolites. **(B)** Volcano plot of the differential intracellular metabolites. **(C)** Volcano plot of the differential extracellular metabolites. **(D)** KEGG enrichment analysis based on intracellular metabolites. **(E)** KEGG enrichment analysis based on extracellular metabolites. *P < 0.05; **P < 0.01; ***P < 0.001.

After data preprocessing and comparative analysis, 970 differential intracellular metabolites were identified between gapN and the control (Figures 4A, B), whereas 1,149 differential extracellular metabolites were identified between gapN_s and control_s (Figures 4A, C). KEGG enrichment analysis based on the differential intracellular metabolites revealed 12 enriched pathways (corrected *p* value < 0.05), and the top 5 pathways were “tryptophan metabolism” (map00380), “biosynthesis of plant secondary metabolites” (map01060), “purine metabolism” (map00230), “phenylpropanoid biosynthesis” (map00940), and “biosynthesis of alkaloids derived from histidine and purine” (map01065) (Figure 4D). KEGG enrichment analysis based on the differential extracellular metabolites revealed 9 enriched pathways (corrected *p* value < 0.05), and the top 5 pathways were “biosynthesis of alkaloids derived from histidine and purine” (map01065), “purine metabolism” (map00230), “tryptophan metabolism” (map00380), “biosynthesis of plant secondary metabolites” (map01060) and “steroid hormone biosynthesis” (map00140) (Figure 4E). The pathways that which were enriched both intracellularly and extracellularly included “biosynthesis of plant secondary metabolites,” “tryptophan metabolism,” “purine metabolism,” “biosynthesis of alkaloids derived from histidine and purine,” and “beta-alanine metabolism.”

### 3.5 Combination of transcriptome and metabolism analyses

Transcriptomics analysis was primarily employed to elucidate the impact of *gapN* expression on ε-PL metabolism; hence, samples were chosen at the 48-h mark. Metabolomics, on the other hand, was primarily utilized to discern alterations in SMs of *S. albulus* influenced by *gapN* expression; thus, samples were selected after 7 days. Despite the disparate sampling timeframes, certain correlations were observed between the data generated in these two experiments. The network diagrams of differential intracellular metabolites and differential genes identified via KEGG enrichment analysis are presented in Supplementary Figures 5A, B, respectively. The analysis revealed enrichment of multiple metabolic pathways in both the transcriptomic and metabolomic data. Notably, “beta-alanine metabolism” metabolic pathway exhibited enrichment in both studies, indicating the influence of *gapN* expression on the metabolism and biosynthesis of amino acids. Given the significance of amino acids as crucial precursors for SM biosynthesis, several metabolic pathways associated with SM biosynthesis were enriched in the metabolomic studies. The metabolic perturbation of *S. albulus* due to *gapN* expression was visualized using the interactive Pathways Explorer v3 (iPath 3).^4^ This visualization was based on KEGG annotations of the transcriptome and intracellular metabolism results, as shown in Supplementary Figure 5C.

### 3.6 Effects of *gapN* expression on the ε-PL metabolic pathway

**FIGURE 5:**
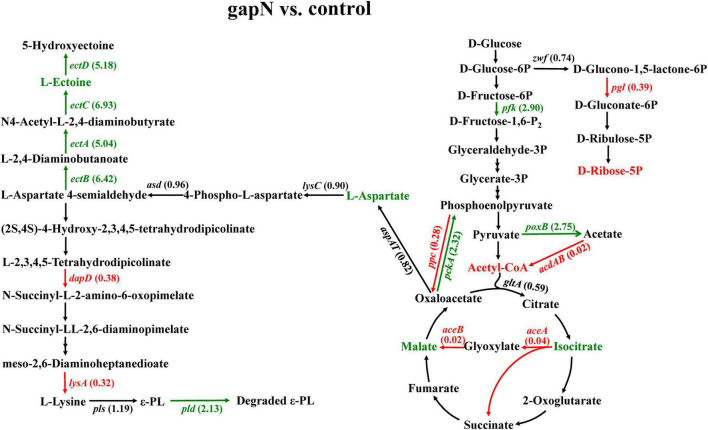
Effects of *gapN* expression on the ε-PL biosynthesis pathway. The green lines, arrows, and gene names represent genes whose expression was significantly upregulated at each step, whereas the red lines, arrows and gene names represent genes whose expression was significantly downregulated. The numbers that follow the gene names are the FCs in gene expression. The green and red metabolite names correspond to the upregulated and downregulated metabolites detected by metabolomics, respectively. *zwf*, glucose-6-phosphate dehydrogenase; *pgl*, 6-phosphogluconolactonase; *pfk*, 6-phosphofructokinase; *ppc*, phosphoenolpyruvate carboxylase; *pckA*, phosphoenolpyruvate carboxykinase; *poxB*, pyruvate oxidase; *acdAB*, acetate-CoA ligase (ADP-forming); *gltA*, citrate synthase; *aceA*, isocitrate lyase; *aceB*, malate synthase A; *lysC*, aspartate kinase; *asd*, aspartate-semialdehyde dehydrogenase; *ectA*, L-2,4-diaminobutyrate acetyltransferase; *ectB*, diaminobutyrate-2-oxoglutarate transaminase; *ectC*, L-ectoine synthase; *ectD*, ectoine hydroxylase; *dapA*, 4-hydroxytetrahydrodipicolinate synthase; *dapD*, 2,3,4,5-tetrahydropyridine-2,6-dicarboxylate N-succinyltransferase; *argD*, acetylornithine aminotransferase; *lysA*, diaminopimelate decarboxylase; *pls*, polylysine synthetase; and *pld*, ε-PL degrading enzyme.

**FIGURE 6:**
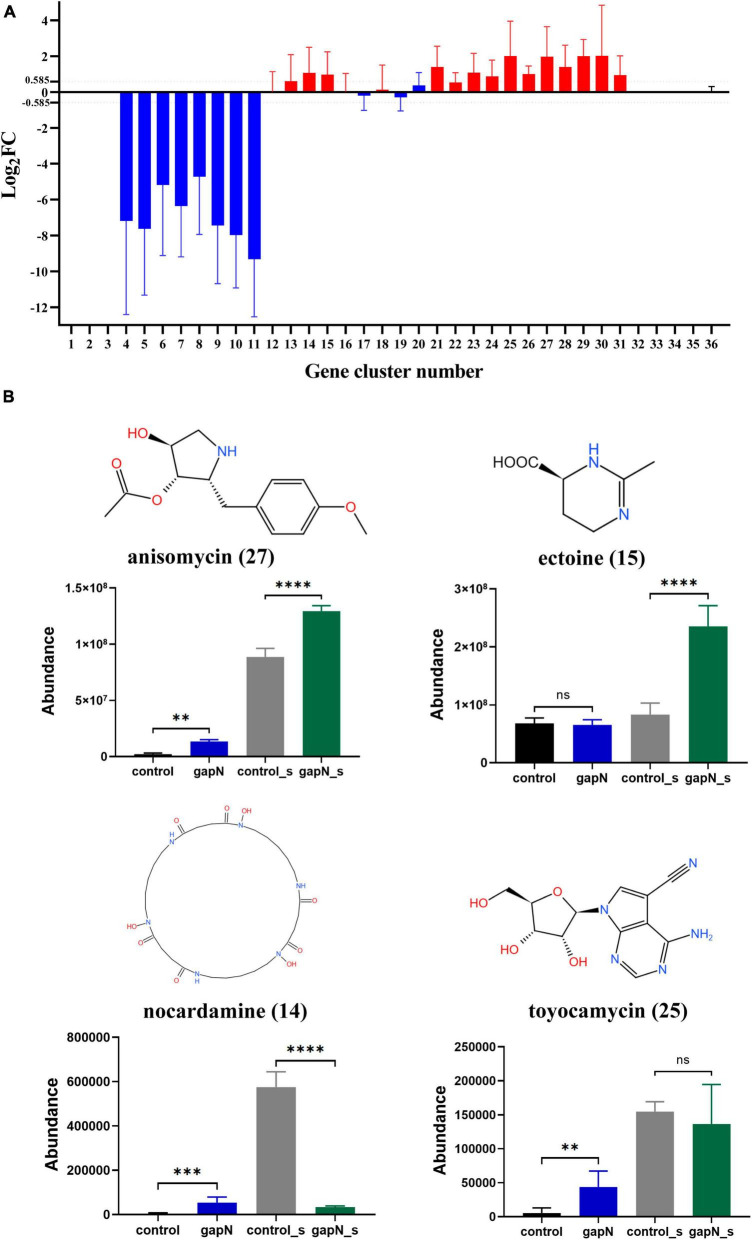
Effects of *gapN* expression on SM-BGC expression and SM biosynthesis in *S. albulus* CICC11022. **(A)** Regulation of the 36 SM-BGCs by *gapN* expression. The numbers on the *x*-axis correspond to the SM-BGC numbers. **(B)** The predicted secondary metabolites of the SM-BGCs that were identified in the metabolomic data. The numbers in parentheses after the metabolite names are the SM-BGC numbers. **P < 0.01; ***P < 0.001; ****P < 0.0001; ns, not significant.

The expression of *gapN* in *S. albulus* resulted in decreased ε-PL production. The effects of *gapN* expression on the ε-PL biosynthesis pathway were then further analyzed via transcriptomic and metabolomic analyses. As shown in Figure 5, the expression of phosphoenolpyruvate carboxylase (*ppc*) and phosphoenolpyruvate carboxykinase (*pckA*) was significantly downregulated and upregulated, respectively, by *gapN* expression. These results indicate that the conversion equilibrium between oxaloacetate (OAA) and phosphoenolpyruvate was disrupted, and the latter was favored. Isocitrate lyase (*aceA*) and malate synthase (*aceB*) were significantly downregulated, indicating that the glyoxylate shunt was negatively affected by *gapN* expression. Interestingly, the intracellular concentration of L-aspartate in the *gapN*-expressing strain greatly increased. Moreover, the expression levels of genes encoding the L-ectoine and hydroxyectoine synthesis pathway (*ectA*, *ectB*, *ectC*, and *ectD*) and the gene encoding pyruvate oxidase (*poxB*) were significantly upregulated by *gapN* expression. These two pathways serve as competitive metabolic pathways for ε-PL biosynthesis. L-Aspartate 4-semialdehyde plays a crucial role as an intermediate metabolite in both the biosynthetic pathways of L-lysine and L-ectoine, as depicted in Figure 5. These two metabolic pathways compete for the utilization of this specific compound. Furthermore, there was a significant increase in the extracellular concentration of L-ectoine (Figure 6B), confirming the competitive relationship between the two pathways. Pyruvate oxidase catalyzes the conversion of pyruvate to acetate, leading to a limited availability of acetyl-CoA. The insufficiency of acetyl-CoA may impact the availability of oxaloacetate, a crucial initial metabolite for L-lysine biosynthesis. Additionally, there was a significant downregulation of 6-phosphogluconolactonase (*pgl*), leading to a reduced intracellular accumulation of D-ribose-5P. This result may be due to the reduced dependence of the strain on the pentose phosphate pathway as a result of the increased intracellular NADPH concentrations. Moreover, 2,3,4,5-tetrahydropyridine-2,6-dicarboxylate N-succinyltransferase (*dapD*) and diaminopimelate decarboxylase (*lysA*) were downregulated, whereas the ε-PL degrading enzyme (*pld*) was upregulated.

To summarize, the alterations in gene expression levels and metabolites resulting from *gapN* expression suggest a potential correlation between the decline in ε-PL production in *S. albulus* and a decrease in intracellular L-lysine availability, an increase in ε-PL degradation, and, notably, a reduction in the intracellular ATP concentration.

### 3.7 Effects of *gapN* expression on SM-BGC expression and SM biosynthesis

A total of 36 SM-BGCs in the *S. albulus* CICC11022 genome were predicted by antiSMASH 7.1.0. These SM-BGCs were numbered 1 to 36 according to their location (Supplementary Figure 6; Supplementary Table 5). To determine the effect of *gapN* expression on each SM-BGC, the average Log_2_FC values for all genes in each gene cluster were calculated. SM-BGCs with an average | log_2_FC| > 0.585 (FC > 1.5) were considered to be significantly affected by *gapN* expression. As shown in Figure 6A, SM-BGCs 1–3 and 32–36 were almost completely silent with or without *gapN* expression, indicating that they cannot be activated by *gapN* expression. Heterologous *gapN* expression resulted in the almost complete silencing of SM-BGCs 4–11. SM-BGCs 13–15, 21, and 23–31 were significantly upregulated by *gapN* expression. In summary, a total of 28 SM-BGCs were affected by *gapN* expression, 13 and 8 of which were significantly up- and downregulated, respectively.

The metabolomic data for the predicted SMs of the 21 SM-BGCs affected by *gapN* expression were then carefully checked. Four metabolites were identified, namely, anisomycin, ectoine, nocardamine, and toyocamycin. The intracellular amounts of anisomycin, toyocamycin, and nocardamine and the extracellular amounts of ectoine in the *gapN*-expressing strain were significantly greater than those in the control strain (Figure 6B). These results are consistent with the changes in the expression levels of the corresponding gene clusters (Figure 6A).

**FIGURE 7:**
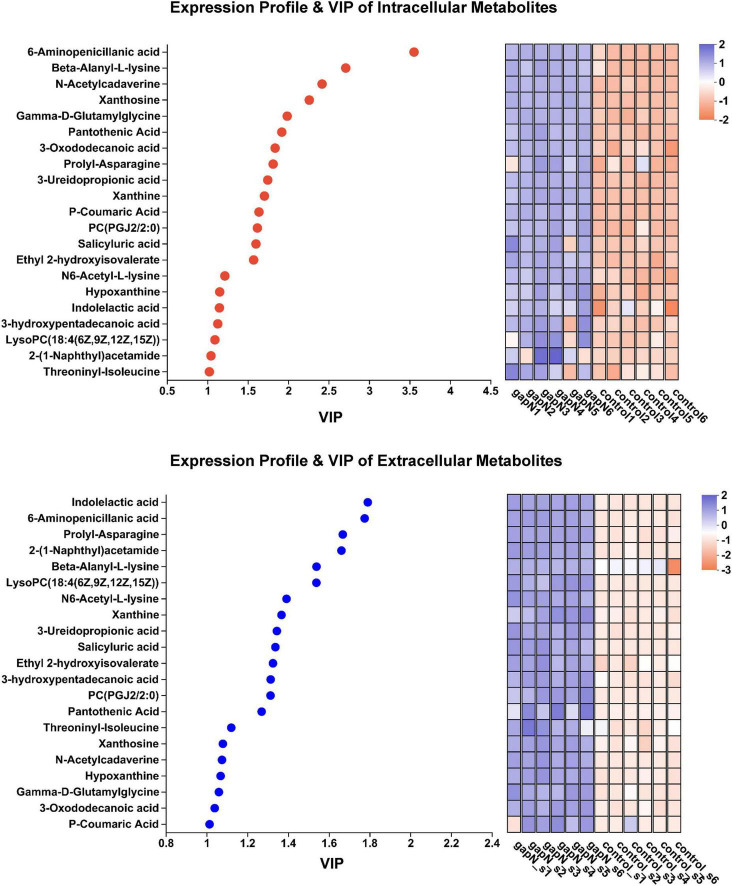
Expression profiles and VIP analysis of the metabolites that were upregulated both intracellularly and extracellularly.

To determine the metabolites that were genuinely influenced by *gapN* expression, we further identified 21 metabolites that exhibited upregulation both intracellularly and extracellularly (Supplementary Table 6). The expression profiles and VIP values of these metabolites are illustrated in Figure 7. Notably, among the metabolites displaying the two highest VIP values both intracellularly and extracellularly was 6-aminopenicillanic acid.

## 4 Discussion

In the present study, the expression of *gapN* was found to have a detrimental effect on the production of ε-PL. One notable distinction between the production of L-lysine and ε-PL is the requirement of ATP for polymerization. The findings of this study indicate that *gapN* expression leads to an increase in the intracellular NADPH concentration and a decrease in the NADH concentration, consequently resulting in a decrease in the intracellular ATP concentration. These alterations in the balance of intracellular cofactors ultimately lead to metabolic shunts in the biosynthesis pathway of ε-PL. The reliance of cells on the pentose phosphate pathway was significantly decreased due to the downregulation of the 6-phosphogluconolactonase gene and the decrease in D-ribose-5P levels in the *gapN* expressing strain (Figure 5). The activation of the L-ectoine biosynthetic pathway was observed as a consequence of gapN expression, leading to the upregulation of the *ectABCD* gene cluster (Figure 5) and subsequent augmentation of L-ectoine synthesis (Figure 6B). In our previous investigation, it was discovered that the activation of the L-ectoine biosynthetic pathway occurs upon deletion of the gene encoding polylysine synthetase (Lian et al., 2022). Consequently, the L-ectoine biosynthetic pathway serves as a competitive metabolic bypass for the ε-PL biosynthetic pathway in *S. albulus* CICC11022. These findings suggest that while *gapN* expression can mitigate the reliance of the L-lysine synthesis pathway on the pentose phosphate pathway, the substantial reduction in ATP supply resulting from *gapN* expression significantly hampers ε-PL production.

The heterologous expression of *gapN* had an unexpected and interesting positive impact on the biosynthesis of bioactive SMs in *S. albulus*. A total of 21 SM-BGCs were significantly affected by *gapN* expression, including three gene clusters associated with antibiotic production, namely, anisomycin, nocardamine, and toyocamycin (Supplementary Table 5; Supplementary Figure 6). Anisomycin is a pyrrolidine alkaloid antibiotic that has notable antifungal, antigenic, antiviral, and antitumor properties (Ye et al., 2019; Quintana et al., 2020). Nocardamine, is a cyclic hydroxamic acid siderophore that has been shown to demonstrate antitumor and antimalarial effects (Mahmud et al., 2022). Finally, toyocamycin, is an adenosine analog that has antitumor and antifungal effects (Pandey et al., 2022; Song et al., 2022). The aforementioned three antibiotics were also identified through metabolomics analysis, and their intracellular levels were positively influenced by *gapN* expression (Figure 6B). Additionally, after 7 days, the concentrations of anisomycin in the fermentation broth of both the *gapN*-expressing strain and the control strain were found to be 155.66 mg/L and 86.24 mg/L, respectively. These findings are consistent with the expected alterations in metabolome profiles. Given the well-established BGCs associated with these antibiotics, it is anticipated that overexpression of their BGCs will further enhance their production. Additionally, 21 metabolites were increased in the *gapN*-expressing strain both intracellularly and extracellularly (Supplementary Table 6). The application of VIP analysis demonstrated that 6-aminopenicillanic acid was among the top two metabolites upregulated (Figure 7). 6-Aminopenicillanic acid, the core β-lactam compound of penicillins, is an important active pharmaceutical intermediate that can be used as the main starting block for the preparation of numerous semisynthetic penicillins (Sawant et al., 2020). With respect to the genome of *S. albulus* CICC11022, we identified a total of 31 penicillin-related genes, including two genes (gene 2388 and gene 3892) encoding penicillin acylases. These finding suggest the potential involvement of these genes in the biosynthesis pathway of 6-aminopenicillanic acid.

In summary, the expression of *gapN* in *S. albulus* significantly increased SM synthesis. This phenomenon could be attributed to an ATP deficit, which is known to trigger the activation of oxidative metabolism to reestablish the energetic balance. This activation is essential for ATP generation concomitant with oxidative stress (Esnault et al., 2017; Lejeune et al., 2022; Apel et al., 2023), and potential detrimental effects should be mitigated by various processes. These processes include the production of specific metabolites with antioxidant properties (Virolle, 2020). Research in the literature has indicated that pantothenic acid (Revuelta et al., 2016), *p*-coumaric acid (Boz, 2015; Boo, 2019), 3-ureidopropionic acid (Noguer et al., 2014) and indolelactic acid (Negatu et al., 2020) indeed exhibit antioxidant properties. Furthermore, the downregulation of *poxB* is expected to lead to a decrease in acetyl-CoA generation and thus to reduced feeding and activity in the TCA cycle. Similarly, the downregulation of *ppc* and upregulation of *pckA* are predicted to lead to a depletion of oxaloacetate from the TCA cycle. Both processes are thought to contribute to the reduction in the activity of the TCA cycle and thus to limit the generation of NADH, whose reoxidation by the respiratory chain is the origin of the generation of ROS/NOS responsible for oxidative stress. Since our present findings point to a significant concern regarding oxidative stress in the *gapN*-expressing strain, we examined the expression of 12 genes classified as involved in the oxidative stress response. Among them, 4 exhibited upregulated expression in the *gapN*-expressing strain (Supplementary Figure 7), including the redox-sensitive transcriptional activator SoxR, a peptide-methionine (*R*)-S-oxide reductase and two catalases, whereas two genes encoding catalase-peroxidase and hydroperoxide resistance protein were significantly downregulated in the *gapN*-expressing strain.

## 5 Conclusion

This study provided a comprehensive view of the metabolic perturbations in *S. albulus* induced by heterologous expression of *gapN*. Moreover, this approach unexpectedly led to the upregulation of the biosynthesis of valuable bioactive SMs. Consequently, this research can serve as a valuable reference for the utilization of genetic engineering techniques to exploit the high metabolic richness of the numerous *Streptomyces* species present on earth for the discovery of novel bioactive SMs whose biosynthesis is directed by the cryptic pathways present in the genomes of these actinobacteria.

## Data availability statement

The original contributions presented in the study are publicly available. This data can be found here: https://www.ncbi.nlm.nih.gov/, SRR20727629 (control) and SRR20727628 (gapN).

## Author contributions

JM: Formal analysis, Investigation, Methodology, Writing—original draft. MZ: Investigation, Methodology, Writing—original draft. WD: Investigation, Writing—original draft. CF: Methodology, Writing—original draft. ZW: Investigation, Writing—original draft. XW: Resources, Visualization, Writing—review and editing. QY: Funding acquisition, Supervision, Writing—review and editing. LK: Funding acquisition, Supervision, Writing—review and editing. JQ: Conceptualization, Writing—review and editing, Funding acquisition, Resources, Supervision.

## Footnotes

1
http://www.majorbio.com/

2
http://www.hmdb.ca/

3
https://metlin.scripps.edu/

4
https://pathways.embl.de/
