# Supplementary material for: Metabolic perturbation of Streptomyces albulus by introducing NADP-dependent glyceraldehyde 3-phosphate dehydrogenase
Source: Front Microbiol. 2024 Jan 24;15:1328321. doi: 10.3389/fmicb.2024.1328321 (PMC10847347; doi:10.3389/fmicb.2024.1328321)
Supplement: Supplementary file 1 [file Data_Sheet_1.pdf]

Supplementary Material

Metabolic perturbation of *Streptomyces albulus* by introducing NADP-dependent glyceraldehyde 3-phosphate dehydrogenase

Jiaqi Mao†, Min Zhang†, Wenjuan Dai, Chenghao Fu, Zhanzhan Wang, Xiuwen Wang, Qingshou Yao, Linghui Kong*, Jiayang Qin*

School of Pharmacy, Binzhou Medical University, Yantai 264003, China

†These authors share first authorship.

*Corresponding author

Email addresses:

L.K.: konglinghui@bzmc.edu.cn

J.Q.: qinjysdu@163.com

Supplementary Table 1 Strains, plasmids, and primers used for construction of the *gapN* expression strain.

Strain and plasmid	Description[a]	Source or reference
Strains		
S. albulus CICC 11022	Wild type strain, transformation host	CICC
S. albulus Q-152	Control strain, *S. albulus* CICC 11022 harboring pSET152	This study
S. albulus Q-gapN	*gapN* heterologous expression strain, *S. albulus* CICC 11022 harboring pSET152-gapN	This study
E. coli ET12567/pUZ8002	*rec*E, *dcm*$^-$, *dam*$^-$, *hsd*S, Cm^r, Tet^r, Str^r, Km^r, non-methylating plasmid donor strain for intergeneric conjugation	56
E. coli Trans5α	General cloning strain	Transgen
Plasmids		
pSET152	5.7 kb, Apr^r, integrative plasmid, *lacZ*α $ori^{pUC19}oriT^{RP4}int\text{-}attP^{\varphi31}aac(3)IV$	56
pSET152-gapN	Apr^r, pSET152 carrying *SP43* promoter, *SR41* RBS, and *gapN* gene	This study
Primers		
gapN-F	GCACGCTCTAGATGTTCACATTCGA ACCGTCTC	This study
gapN-R	GACGACGGATCCTTACTTGATGTCG AAGACGAC	This study
RT-gapN-F	TGGCGCTCCCTGTCCTACA	This study
RT-gapN-R	AGCCCTTGGCGACCTCCTTG	This study

[a]Cm, chloramphenicol; Tet, tetracycline; Str, streptomycin; Km, kanamycin; Apr, apramycin.

Supplementary Table 2 Primers used for qRT-PCR analysis to verify the RNA sequencing data.

Gene	Description	Primers (5′→3′)	Length
Gene3819	hypothetical protein	CTCCAACTCCATCCGCAACT<br>GTAGGGCTCTTCGCTGTTGA	121bp
Gene5145	hypothetical protein	TGGCACTGAGACACCGAATC<br>GTGTCGCCGCAGTCCTATC	76bp
Gene6088	3-isopropylmalate dehydrogenase	TCGACCACTTCGTCAACCTG<br>GACGAAGTCGATGTCGGGG	86bp
Gene7261	7-cyano-7-deazaguanine synthase	GGCGACCACTTCATCTACCCG<br>CGTGGCGATGTCGGTCTTGG	141bp
Gene8171	hypothetical protein	GCTCACCAACTTCGGCCACTT<br>GGTGCAACGACAGTCCCTTGAC	71bp
Gene6380	RNA polymerase sigma factor, *hrdB* (the reference gene)	CTGACCAGATTCCGCCAACCC<br>GCCTCTGCGGCACTGACCAT	100 bp

Supplementary Table 3 Verification of the RNA-sequencing data by qRT-PCR.

Gene ID	Description	Fold change (gapN vs. control)	
		RNA-seq	qRT-PCR
Gene3819	hypothetical protein	0.278	0.418
Gene5145	hypothetical protein	0.004	0.4
Gene6088	3-isopropylmalate dehydrogenase	0.275	0.53
Gene7261	7-cyano-7-deazaguanine synthase	34.6	1.71
Gene8171	hypothetical protein	102.3	2.81

Supplementary Table 4 KEGG annotation of the DEGs.

Gene id	Gene description	control tpm	gapN tpm	KO id	KO name	Pathway id	Pathway definition
gene0439	hypothetical protein	15	0	K00467	------	map00620	Pyruvate metabolism
gene0440	hypothetical protein	36	0	K04093	pheA1	map00400	Phenylalanine, tyrosine and tryptophan biosynthesis
gene0444	hypothetical protein	38	0	K00648	fabH	map00061	Fatty acid biosynthesis
gene0445	hypothetical protein	86	0	K00648	fabH	map00061	Fatty acid biosynthesis
gene0451	hypothetical protein	73	0	K00441	frhB	map00680	Methane metabolism
gene0452	class III aminotransferase	32	0	K07250	gabT	map00310;map00640;map00650;map00250;map00280;map00410	Lysine degradation;Propanoate metabolism;Butanoate metabolism;Alanine, aspartate and glutamate metabolism;Valine, leucine and isoleucine degradation;beta-Alanine metabolism
gene0457	cardiolipin synthase	51	0	K06131	clsA_B	map00564	Glycerophospholipid metabolism
gene0463	alcohol dehydrogenase	4	0	K13953	adhP	map00071;map00980;map00626;map00625;map00620;map00010;map00830;map00350;map00982	Fatty acid degradation;Metabolism of xenobiotics by cytochrome P450;Naphthalene degradation;Chloroalkane and chloroalkene degradation;Pyruvate metabolism;Glycolysis / Gluconeogenesis;Retinol metabolism;Tyrosine metabolism;Drug metabolism - cytochrome P450
gene0464	asparagine synthetase B	48	0	K01953	asnB	map00250	Alanine, aspartate and glutamate metabolism
gene0470	putative PLP-dependent enzyme possibly involved in cell wall biogenesis	183	0	K13010	per	map00520;map00541	Amino sugar and nucleotide sugar metabolism;O-Antigen nucleotide sugar biosynthesis
gene0474	3-hydroxyisobutyrate dehydrogenase	115	0	K00020	mmsB	map00280	Valine, leucine and isoleucine degradation
gene0478	catalase-peroxidase	112	0	K03782	katG	map00940;map00983;map00380;map00360	Phenylpropanoid biosynthesis;Drug metabolism - other enzymes;Tryptophan metabolism;Phenylalanine metabolism
gene0484	short-chain dehydrogenase/reductase	14	0	K00059	fabG	map00780;map00061;map00333	Biotin metabolism;Fatty acid biosynthesis;Prodigiosin biosynthesis
gene0487	UDP-glucose 6-dehydrogenase	5	0	K00012	ugd	map00520;map00040;map00541;map00053	Amino sugar and nucleotide sugar metabolism;Pentose and glucuronate interconversions;O-Antigen nucleotide sugar biosynthesis;Ascorbate and aldarate metabolism
gene0491	phosphogluconate dehydrogenase (NADP(+)-dependent, decarboxylating)	0	0	K00033	gnd	map00480;map00030	Glutathione metabolism;Pentose phosphate pathway
gene0503	adenosine deaminase	9	0	K18286	add	map00130	Ubiquinone and other terpenoid-quinone biosynthesis
gene0523	cytochrome P450	15	0	K21146	------	map00100	Steroid biosynthesis
gene0525	hypothetical protein	0	0	K01921	ddl	map00550;map01502	Peptidoglycan biosynthesis;Vancomycin resistance
gene0526	hypothetical protein	1	0	K01046	lip	map00561	Glycerolipid metabolism
gene0538	protocatechuate 3,4-dioxygenase subunit beta	1	0	K00449	pcaH	map00362;map00624	Benzoate degradation;Polycyclic aromatic hydrocarbon degradation
gene0539	3-oxoacid CoA-transferase subunit B	1	0	K01029	scoB	map00280;map00650	Valine, leucine and isoleucine degradation;Butanoate metabolism
gene0562	xanthine dehydrogenase molybdopterin binding subunit	3	0	K13482	xdhB	map00230	Purine metabolism
gene0563	dehydrogenase	5	0	K13481	xdhA	map00230	Purine metabolism
gene0579	3-oxoacyl-ACP reductase	2	0	K00059	fabG	map00780;map00061;map00333	Biotin metabolism;Fatty acid biosynthesis;Prodigiosin biosynthesis
gene0592	cyanate hydratase	1	0	K01725	cynS	map00910	Nitrogen metabolism
gene0603	molecular chaperone HtpG	36	0	K04079	htpG	map05207;map05215;map04657;map05200;map04914;map04659;map04151;map04915;map04621;map04626;map05418;map05132;map05417;map04217;map04612;map04141	Chemical carcinogenesis - receptor activation;Prostate cancer;IL-17 signaling pathway;Pathways in cancer;Progesterone-mediated oocyte maturation;Th17 cell differentiation;PI3K-Akt signaling pathway;Estrogen signaling pathway;NOD-like receptor signaling pathway;Plant-pathogen interaction;Fluid shear stress and atherosclerosis;Salmonella infection;Lipid and atherosclerosis;Necroptosis;Antigen processing and presentation;Protein processing in endoplasmic reticulum
gene0613	preprotein translocase subunit SecD	1	0	K12257	secDF	map02024;map03070;map03060	Quorum sensing;Bacterial secretion system;Protein export
gene0622	hypothetical protein	0	0	K01485	codA	map00240;map00330	Pyrimidine metabolism;Arginine and proline metabolism
gene0623	hypothetical protein	1	0	K05881	dhaM	map00561	Glycerolipid metabolism
gene0625	dihydroxyacetone kinase, L subunit	1	0	K05879	dhaL	map00561	Glycerolipid metabolism
gene0626	4-hydroxyphenylpyruvate dioxygenase	0	0	K16421	hmaS	map00261;map01055	Monobactam biosynthesis;Biosynthesis of vancomycin group antibiotics
gene0627	alpha-hydroxy-acid oxidizing enzyme	0	0	K16422	hmo	map00261;map01055	Monobactam biosynthesis;Biosynthesis of vancomycin group antibiotics
gene0629	aminotransferase	2	0	K16423	hpgT	map00261;map01055	Monobactam biosynthesis;Biosynthesis of vancomycin group antibiotics
gene0631	prephenate dehydrogenase	12	0	K04517	tyrA2	map00400;map00401	Phenylalanine, tyrosine and tryptophan biosynthesis;Novobiocin biosynthesis
gene0632	isomerase	27	0	K01826	hpaF	map00350	Tyrosine metabolism
gene0633	hypothetical protein	5	0	K01512	acyP	map00627;map00620	Aminobenzoate degradation;Pyruvate metabolism
gene0663	UDP-N-acetylglucosamine--N-acetylmuramyl-(pentapeptide) pyrophosphoryl-undecaprenol N-acetylglucosamine transferase	2	0	K02563	murG	map04112;map00550;map01502	Cell cycle - Caulobacter;Peptidoglycan biosynthesis;Vancomycin resistance
gene0664	polyprenyl synthetase	9	0	K13787	idsA	map00900	Terpenoid backbone biosynthesis
gene0691	acyl transferase domain-containing protein	0	0	K00645	fabD	map00061;map00333	Fatty acid biosynthesis;Prodigiosin biosynthesis
gene0692	hypothetical protein	0	0	K00232	------	map00640;map00592;map03320;map04024;map0	Propanoate metabolism;alpha-Linolenic acid metabolism;PPAR signaling pathway;cAMP signaling

						1040;map00071;map04146;map00410	pathway;Biosynthesis of unsaturated fatty acids;Fatty acid degradation;Peroxisome;beta-Alanine metabolism
gene0695	3-oxoacyl-ACP synthase	0	0	K00648	fabH	map00061	Fatty acid biosynthesis
gene0699	NAD(P)-dependent alcohol dehydrogenase	6	0	K00055	------	map00360;map00622;map00623;map00350	Phenylalanine metabolism;Xylene degradation;Toluene degradation;Tyrosine metabolism
gene0701	3-octaprenyl-4-hydroxybenzoate carboxy-lyase	0	0	K03186	ubiX	map00627;map00130;map00940	Aminobenzoate degradation;Ubiquinone and other terpenoid-quinone biosynthesis;Phenylpropanoid biosynthesis
gene0711	tartrate dehydrogenase	2	0	K07246	ttuC	map00630;map00650	Glyoxylate and dicarboxylate metabolism;Butanoate metabolism
gene0723	serine hydrolase	7	0	K17836	penP	map00311;map01501	Penicillin and cephalosporin biosynthesis;beta-Lactam resistance
gene0725	class A beta-lactamase	1	0	K17836	penP	map00311;map01501	Penicillin and cephalosporin biosynthesis;beta-Lactam resistance
gene0738	acetolactate synthase	0	0	K12253	aruI	map00330	Arginine and proline metabolism
gene0739	glutamate dehydrogenase	1	0	K00261	gdhA	map04964;map00220;map00250;map00910;map04217	Proximal tubule bicarbonate reclamation;Arginine biosynthesis;Alanine, aspartate and glutamate metabolism;Nitrogen metabolism;Necroptosis
gene0813	tyrosine phenol-lyase	1	0	K01668	------	map00350	Tyrosine metabolism
gene0826	hypothetical protein	11	0	K01179	------	map00500;map02020	Starch and sucrose metabolism;Two-component system
gene0893	carbonic anhydrase	2	0	K01673	cynT	map00910	Nitrogen metabolism
gene0903	cytochrome ubiquinol oxidase subunit I	749	0	K02274	coxA	map00190	Oxidative phosphorylation
gene0915	oxidoreductase iron-sulfur-binding subunit	97	0	K13483	yagT	map00230	Purine metabolism
gene0916	oxidoreductase	31	0	K11178	yagS	map00230	Purine metabolism
gene0917	oxidoreductase	24	0	K11177	yagR	map00230	Purine metabolism
gene0931	4-hydroxybenzoyl-CoA thioesterase	1	0	K01075	------	map00362;map00130	Benzoate degradation;Ubiquinone and other terpenoid-quinone biosynthesis
gene0936	potassium transporter KtrB	1	0	K01547	kdpB	map02020	Two-component system
gene0943	hypothetical protein	1	0	K01312	------	map04972;map05164;map04080;map04974	Pancreatic secretion;Influenza A;Neuroactive ligand-receptor interaction;Protein digestion and absorption
gene0952	mycothiol-dependent maleylpyruvate isomerase	1	0	K16163	------	map00350	Tyrosine metabolism
gene0962	hypothetical protein	8	0	K18896	gsmt	map00260	Glycine, serine and threonine metabolism
gene0986	alcohol dehydrogenase	3	0	K01607	pcaC	map00362	Benzoate degradation
gene0998	dihydrolipoyl dehydrogenase	1	0	K00382	lpd	map00630;map00310;map00640;map00620;map00010;map00260;map00020;map00280;map00380	Citrate cycle (TCA cycle);Valine, leucine and isoleucine degradation;Tryptophan metabolism
gene1004	D-alanine--D-alanine ligase	1	0	K01921	ddl	map00550;map01502	Peptidoglycan biosynthesis;Vancomycin resistance
gene1005	hydroxymethylbilane synthase	39	0	K01749	hemC	map00860	Porphyrin and chlorophyll metabolism
gene1015	aspartate racemase	10	0	K01779	racD	map00250;map01054	Alanine, aspartate and glutamate metabolism;Nonribosomal peptide structures
gene1034	hypothetical protein	1	0	K14188	dltC	map05150;map02020;map01503	Cationic antimicrobial peptide (CAMP) resistance
gene1079	MbtH-like protein	3	0	K05375	mbtH	map00261	Monobactam biosynthesis
gene1105	chemotaxis protein CheY	3	0	K00384	trxB	map00450	Selenocompound metabolism
gene1115	ferredoxin reductase	2	0	K00529	hcaD	map00360;map00071	Phenylalanine metabolism;Fatty acid degradation
gene1127	glycoside hydrolase family 15	0	0	K22934	------	map00500	Starch and sucrose metabolism
gene1133	alkene reductase	3	0	K10680	nemA	map00633	Nitrotoluene degradation
gene1140	carbonyl reductase	0	0	K00079	------	map00590;map05208;map05204;map00790;map00980	Arachidonic acid metabolism;Chemical carcinogenesis - reactive oxygen species;Chemical carcinogenesis - DNA adducts;Folate biosynthesis;Metabolism of xenobiotics by cytochrome P450
gene1151	ABC transporter ATP-binding protein	0	0	K16013	cydD	map02010	ABC transporters
gene1163	nitrile hydratase subunit beta	0	0	K20807	nthB	map00627;map00364;map00380;map00643	Aminobenzoate degradation;Fluorobenzoate degradation;Tryptophan metabolism;Styrene degradation
gene1164	nitrile hydratase subunit alpha	1	0	K01721	nthA	map00627;map00364;map00380;map00643	Aminobenzoate degradation;Fluorobenzoate degradation;Tryptophan metabolism;Styrene degradation
gene1167	citrate synthase/methylcitrate synthase	1	0	K01647	gltA	map00020;map00630	Citrate cycle (TCA cycle);Glyoxylate and dicarboxylate metabolism
gene1168	2-methylisocitrate lyase	0	0	K03417	prpB	map00640	Propanoate metabolism
gene1169	2-methylcitrate dehydratase	1	0	K01720	prpD	map00640	Propanoate metabolism
gene1172	isocitrate lyase	0	0	K01637	aceA	map00630	Glyoxylate and dicarboxylate metabolism
gene1173	malate synthase A	1	0	K01638	aceB	map00630;map00620	Glyoxylate and dicarboxylate metabolism;Pyruvate metabolism
gene1174	3-hydroxybutyryl-CoA dehydrogenase	0	0	K00074	paaH	map00362;map00360;map00650	Benzoate degradation;Phenylalanine metabolism;Butanoate metabolism
gene1179	asparagine synthetase B	49	0	K01953	asnB	map00250	Alanine, aspartate and glutamate metabolism
gene1189	dehydrogenase	8	0	K00059	fabG	map00780;map00061;map00333	Biotin metabolism;Fatty acid biosynthesis;Prodigiosin biosynthesis
gene1212	tautomerase	13	0	K01821	praC	map00362;map00622;map00621	Benzoate degradation;Xylene degradation;Dioxin degradation
gene1222	pyridoxal-5'-phosphate-dependent protein subunit beta	0	0	K22847	cysK2	map00270	Cysteine and methionine metabolism
gene1226	NAD(+) kinase	1	0	K00858	ppnK	map00760	Nicotinate and nicotinamide metabolism
gene1232	multicopper oxidase	0	0	K22552	mmcO	map00860	Porphyrin and chlorophyll metabolism
gene1236	shikimate dehydrogenase	0	0	K13832	aroDE	map00400	Phenylalanine, tyrosine and tryptophan biosynthesis
gene1243	enoyl-CoA hydratase	0	0	K07546	bbsH	map00623	Toluene degradation
gene1261	GDP-mannose 6-dehydrogenase	66	0	K00066	algD	map00520;map00051;map02020	Amino sugar and nucleotide sugar metabolism;Fructose and mannose metabolism;Two-component system

gene1265	asparagine synthetase B	24	0	K01953	asnB	map00250	Alanine, aspartate and glutamate metabolism
gene1272	transferase	36	0	K00978	rfbF	map00520;map00500;map00541	Amino sugar and nucleotide sugar metabolism;Starch and sucrose metabolism;O-Antigen nucleotide sugar biosynthesis
gene1279	dTDP-4-dehydrorhamnose 3,5-epimerase	14	0	K01790	rfbC	map00521;map00523;map00541	Streptomycin biosynthesis;Polyketide sugar unit biosynthesis;O-Antigen nucleotide sugar biosynthesis
gene1280	glutamate-1-semialdehyde 2,1-aminomutase	19	0	K01845	hemL	map00860	Porphyrin and chlorophyll metabolism
gene1295	sulfurtransferase	0	0	K01069	gloB	map00620	Pyruvate metabolism
gene1302	(2Fe-2S)-binding protein	3	0	K19982	prnD	map00404	Staurosporine biosynthesis
gene1303	(2Fe-2S)-binding protein	5	0	K19982	prnD	map00404	Staurosporine biosynthesis
gene1304	farnesyl-diphosphate synthase	13	0	K13789	------	map00900	Terpenoid backbone biosynthesis
gene1325	2,3-dihydroxybenzoate-glycine-threonine trimeric ester bacillibactin synthetase	1	0	K02364	entF	map01053	Biosynthesis of siderophore group nonribosomal peptides
gene1333	acetyl-CoA synthetase	1	0	K01895	acs	map00630;map00640;map00720;map00620;map00680;map00010	Glyoxylate and dicarboxylate metabolism;Propanoate metabolism;Carbon fixation pathways in prokaryotes;Pyruvate metabolism;Methane metabolism;Glycolysis / Gluconeogenesis
gene1335	TrpB-like pyridoxal-phosphate dependent enzyme	2	0	K06001	trpB	map00400;map00260	Phenylalanine, tyrosine and tryptophan biosynthesis;Glycine, serine and threonine metabolism
gene1356	hypothetical protein	0	0	K01745	hutH	map00340	Histidine metabolism
gene1377	dehydrogenase	19	0	K03885	ndh	map00190	Oxidative phosphorylation
gene1384	polyketide synthase	13	0	K09458	fabF	map00780;map00061	Biotin metabolism;Fatty acid biosynthesis
gene1386	MbtH domain-containing protein	14	0	K05375	mbtH	map00261	Monobactam biosynthesis
gene1387	hypothetical protein	22	0	K00604	fmt	map00670;map00970	One carbon pool by folate;Aminoacyl-tRNA biosynthesis
gene1403	hypothetical protein	5	0	K14340	------	map00571	Lipoarabinomannan (LAM) biosynthesis
gene1404	D-alanyl-D-alanine carboxypeptidase	19	0	K07258	dacC	map00550	Peptidoglycan biosynthesis
gene1430	class A beta-lactamase	3	0	K17836	penP	map00311;map01501	Penicillin and cephalosporin biosynthesis;beta-Lactam resistance
gene1432	serine hydrolase	2	0	K17836	penP	map00311;map01501	Penicillin and cephalosporin biosynthesis;beta-Lactam resistance
gene1436	5-methyltetrahydropteroyltriglutamate--homocysteine methyltransferase	30	0	K00549	metE	map00450;map00270	Selenocompound metabolism;Cysteine and methionine metabolism
gene1446	tRNA-specific adenosine deaminase	1	0	K01487	guaD	map00230	Purine metabolism
gene1448	hypothetical protein	0	0	K12528	xdhD	map00450	Selenocompound metabolism
gene1467	cytochrome P450	0	0	K20420	ncsB3	map01059	Biosynthesis of enediyne antibiotics
gene1474	hydrolase	8	0	K01207	nagZ	map00520;map01501;map00531	Amino sugar and nucleotide sugar metabolism;beta-Lactam resistance;Glycosaminoglycan degradation
gene1528	adenosine deaminase	94	0	K01488	add	map05340;map00230	Primary immunodeficiency;Purine metabolism
gene1530	glyceraldehyde-3-phosphate dehydrogenase	12	0	K00134	gapA	map00710;map00010;map05010;map05130;map05132;map05415;map04066	Carbon fixation in photosynthetic organisms;Glycolysis / Gluconeogenesis;Alzheimer disease;Pathogenic Escherichia coli infection;Salmonella infection;Diabetic cardiomyopathy;HIF-1 signaling pathway
gene1532	aspartate ammonia-lyase	2	0	K01744	aspA	map00250	Alanine, aspartate and glutamate metabolism
gene1541	bifunctional 3-phenylpropionate/cinnamic acid dioxygenase ferredoxin subunit	4	0	K05710	hcaC	map00360	Phenylalanine metabolism
gene1558	cholesterol oxidase	84	0	K03333	choD	map00984	Steroid degradation
gene1559	FscMI	28	0	K16388	amphDI	map01052	Type I polyketide structures
gene1560	aminotransferase	49	0	K13010	per	map00520;map00541	Amino sugar and nucleotide sugar metabolism;O-Antigen nucleotide sugar biosynthesis
gene1561	cytochrome P450	29	0	K16389	amphN	map01052	Type I polyketide structures
gene1563	type I polyketide synthase	8	0	K16391	pimS0	map01052	Type I polyketide structures
gene1565	type I polyketide synthase	9	0	K19203	pimS1	map01052	Type I polyketide structures
gene1567	cytochrome P450	5	0	K16390	amphL	map01052	Type I polyketide structures
gene1568	type I polyketide synthase	10	0	K19206	pimS4	map01052	Type I polyketide structures
gene1569	type I polyketide synthase	8	0	K19205	pimS3	map01052	Type I polyketide structures
gene1570	type I polyketide synthase	7	0	K19204	pimS2	map01052	Type I polyketide structures
gene1575	GDP-mannose 4,6-dehydratase	23	0	K01711	gmd	map00520;map00051;map00541	Amino sugar and nucleotide sugar metabolism;Fructose and mannose metabolism;O-Antigen nucleotide sugar biosynthesis
gene1576	multidrug ABC transporter ATP-binding protein	11	0	K18887	efrA	map02010	ABC transporters
gene1577	hypothetical protein	8	0	K18888	efrB	map02010	ABC transporters
gene1578	crotonyl-CoA carboxylase/reductase	12	0	K17829	ccrA	map00650	Butanoate metabolism
gene1599	glutamate decarboxylase	50	0	K01580	gadB	map00430;map02024;map00650;map00250;map04940;map04727;map00410	Taurine and hypotaurine metabolism;Quorum sensing;Butanoate metabolism;Alanine, aspartate and glutamate metabolism;Type I diabetes mellitus;GABAergic synapse;beta-Alanine metabolism
gene1608	bifunctional 5,10-methylene-tetrahydrofolate dehydrogenase/5,10-methylene-	21	0	K01491	folD	map00720;map00670	Carbon fixation pathways in prokaryotes;One carbon pool by folate

gene1616	tetrahydrofolate cyclohydrolase Leucyl aminopeptidase	2	5	K18028	nicX	map00760	Nicotinate and nicotinamide metabolism
gene1617	fumarate reductase/succinate dehydrogenase flavoprotein domain-containing protein	1	3	K00480	------	map00626;map00624;map00621	Naphthalene degradation;Polycyclic aromatic hydrocarbon degradation;Dioxin degradation
gene1632	hypothetical protein	59	26	K17686	copA	map04978;map04016;map01524	Mineral absorption;MAPK signaling pathway - plant;Platinum drug resistance
gene1668	hypothetical protein	1	3	K01912	paaK	map00360;map05111	Phenylalanine metabolism;Biofilm formation - Vibrio cholerae
gene1739	short-chain dehydrogenase	2	6	K00059	fabG	map00780;map00061;map00333	Biotin metabolism;Fatty acid biosynthesis;Prodigiosin biosynthesis
gene1747	epoxide hydrolase	3	8	K21159	sgcF	map01059	Biosynthesis of enediyne antibiotics
gene1765	hypothetical protein	1	0	K03651	cpdA	map02025;map00230	Biofilm formation - Pseudomonas aeruginosa;Purine metabolism
gene1784	4-hydroxy-tetrahydrodipicolinate synthase	1	21	K01714	dapA	map00300;map00261	Lysine biosynthesis;Monobactam biosynthesis
gene1895	group 1 glycosyl transferase	1	0	K14335	pimC	map00571	Lipoarabinomannan (LAM) biosynthesis
gene1909	enoyl-CoA hydratase	2	1	K01692	paaF	map00310;map00640;map00281;map00650;map00627;map00903;map00930;map00380;map00362;map00360;map00071;map00280;map00410	Lysine degradation;Propanoate metabolism;Geraniol degradation;Butanoate metabolism;Aminobenzoate degradation;Limonene and pinene degradation;Caprolactam degradation;Tryptophan metabolism;Benzoate degradation;Phenylalanine metabolism;Fatty acid degradation;Valine, leucine and isoleucine degradation;beta-Alanine metabolism
gene1911	chorismate mutase	2	1	K04093	pheA1	map00400	Phenylalanine, tyrosine and tryptophan biosynthesis
gene1918	long-chain-fatty-acid--CoA ligase	2	1	K01897	fadD	map04714;map02024;map03320;map04920;map00061;map00071;map04146;map04216	Thermogenesis;Quorum sensing;PPAR signaling pathway;Adipocytokine signaling pathway;Fatty acid biosynthesis;Fatty acid degradation;Peroxisome;Ferroptosis
gene1931	phosphoketolase	0	3	K01621	xfp	map00030;map00710	Pentose phosphate pathway;Carbon fixation in photosynthetic organisms
gene1944	adenosine deaminase	1	0	K01488	add	map05340;map00230	Primary immunodeficiency;Purine metabolism
gene1960	aldehyde dehydrogenase	3	1	K00128	------	map00310;map00561;map00280;map00981;map00903;map00625;map00330;map00053;map00380;map00010;map00620;map00071;map00340;map00770;map00410	Lysine degradation;Glycerolipid metabolism;Valine, leucine and isoleucine degradation;Insect hormone biosynthesis;Limonene and pinene degradation;Chloroalkane and chloroalkene degradation;Arginine and proline metabolism;Ascorbate and aldarate metabolism;Tryptophan metabolism;Glycolysis / Gluconeogenesis;Pyruvate metabolism;Fatty acid degradation;Histidine metabolism;Pantothenate and CoA biosynthesis;beta-Alanine metabolism
gene1962	hypothetical protein	3	1	K00303	soxB	map00260	Glycine, serine and threonine metabolism
gene1966	substrate-binding protein	1	7	K10227	smoE	map02010	ABC transporters
gene1967	ABC transporter permease	1	6	K10228	smoF	map02010	ABC transporters
gene1968	mannitol ABC transporter permease	0	4	K10229	smoG	map02010	ABC transporters
gene2173	adenine phosphoribosyltransferase	82	44	K00759	apt	map00230	Purine metabolism
gene2181	glutamine amidotransferase subunit PdxT	17	45	K08681	pdxT	map00750	Vitamin B6 metabolism
gene2182	pyridoxal biosynthesis lyase PdxS	84	213	K06215	pdxS	map00750	Vitamin B6 metabolism
gene2219	ABC transporter substrate-binding protein	60	27	K02073	metQ	map02010	ABC transporters
gene2220	binding-protein-dependent transport systems inner membrane component	25	10	K02072	metI	map02010	ABC transporters
gene2226	1-acyl-sn-glycerol-3-phosphate acyltransferase	60	31	K00655	plsC	map00564;map00561	Glycerophospholipid metabolism;Glycerolipid metabolism
gene2227	bifunctional metallophosphatase/5'-nucleotidase	0	4	K01081	------	map00240;map00230;map00760	Pyrimidine metabolism;Purine metabolism;Nicotinate and nicotinamide metabolism
gene2281	glutamine synthetase	13	5	K01915	glnA	map00630;map00220;map00910;map02020;map00250;map04727;map04724;map04217	Glyoxylate and dicarboxylate metabolism;Arginine biosynthesis;Nitrogen metabolism;Two-component system;Alanine, aspartate and glutamate metabolism;GABAergic synapse;Glutamatergic synapse;Necroptosis
gene2291	glycine/betaine ABC transporter substrate-binding protein	2	6	K02001	proW	map02010	ABC transporters
gene2292	glycine/betaine ABC transporter ATP-binding protein	1	4	K02000	proV	map02010	ABC transporters
gene2344	mandelate racemase	0	1	K18334	fucD	map00051	Fructose and mannose metabolism
gene2438	asparagine synthetase B	89	308	K01953	asnB	map00250	Alanine, aspartate and glutamate metabolism
gene2520	L-2,4-diaminobutyrate decarboxylase	8	53	K13745	ddc	map00260	Glycine, serine and threonine metabolism
gene2561	cyclic pyranopterin phosphate synthase MoaA	31	70	K03639	moaA	map04122;map00790	Sulfur relay system;Folate biosynthesis
gene2606	L-2,4-diaminobutyrate acetyltransferase	11	63	K06718	ectA	map00260	Glycine, serine and threonine metabolism

gene2607	diaminobutyrate--2-oxoglutarate transaminase	12	88	K00836	ectB	map00260	Glycine, serine and threonine metabolism
gene2608	L-ectoine synthase	17	134	K06720	ectC	map00260	Glycine, serine and threonine metabolism
gene2609	ectoine hydroxylase	25	145	K10674	ectD	map00260	Glycine, serine and threonine metabolism
gene2629	2,3,4,5-tetrahydropyridine-2,6-dicarboxylate N-succinyltransferase	91	39	K00674	dapD	map00300	Lysine biosynthesis
gene2651	6-phosphogluconolactonase	64	28	K01057	pgl	map00030	Pentose phosphate pathway
gene2675	hypothetical protein	36	19	K16163	------	map00350	Tyrosine metabolism
gene2729	aminopeptidase N	11	4	K01256	pepN	map00480	Glutathione metabolism
gene2731	beta-galactosidase	0	0	K12308	bgaB	map00052	Galactose metabolism
gene2747	glutamate synthase subunit beta	128	71	K00266	gltD	map00250;map00910	Alanine, aspartate and glutamate metabolism;Nitrogen metabolism
gene2813	cell division protein	10	22	K03587	ftsI	map00550;map01501	Peptidoglycan biosynthesis;beta-Lactam resistance
gene2816	plant-type carbonic anhydrase	42	96	K01673	cynT	map00910	Nitrogen metabolism
gene2844	2-hydroxyacid dehydrogenase	3	7	K03778	ldhA	map00620	Pyruvate metabolism
gene2927	glutamate--ammonia ligase	88	36	K01915	glnA	map00630;map00220;map00910;map02020;map00250;map04727;map04724;map04217	Glyoxylate and dicarboxylate metabolism;Arginine biosynthesis;Nitrogen metabolism;Two-component system;Alanine, aspartate and glutamate metabolism;GABAergic synapse;Glutamatergic synapse;Necroptosis
gene3000	heme oxygenase	91	48	K21480	pbsA1	map00860	Porphyrin and chlorophyll metabolism
gene3066	cytochrome C oxidase subunit I	34	4	K02274	coxA	map00190	Oxidative phosphorylation
gene3324	gamma-glutamyl kinase	23	11	K00931	proB	map00330;map00332	Arginine and proline metabolism;Carbapenem biosynthesis
gene3430	3D-(3,5/4)-trihydroxycyclohexane-1,2-dione hydrolase	15	7	K03336	iolD	map00562	Inositol phosphate metabolism
gene3431	5-deoxy-glucuronate isomerase	17	7	K03337	iolB	map00562	Inositol phosphate metabolism
gene3467	acyl-CoA thioesterase II	5	3	K10805	tesB	map01040	Biosynthesis of unsaturated fatty acids
gene3468	acyl-CoA dehydrogenase	13	7	K00249	acd	map00280;map03320;map00071	Valine, leucine and isoleucine degradation;PPAR signaling pathway;Fatty acid degradation
gene3470	acetyl-CoA carboxylase subunit beta	12	5	K01969	------	map00280	Valine, leucine and isoleucine degradation
gene3471	acetyl/propionyl-CoA carboxylase subuit alpha	10	4	K11263	bccA	map00630;map00280;map00640;map00620;map00061	Glyoxylate and dicarboxylate metabolism;Valine, leucine and isoleucine degradation;Propanoate metabolism;Pyruvate metabolism;Fatty acid biosynthesis
gene3472	hydroxymethylglutaryl-CoA lyase	9	5	K01640	hmgL	map04146;map00280;map00281;map00650	Peroxisome;Valine, leucine and isoleucine degradation;Geraniol degradation;Butanoate metabolism
gene3566	phosphotransferase system IIC component	12	4	K02804	nagE	map00520;map02060	Phosphotransferase system (PTS)
gene3593	oxidoreductase	5	11	K00104	glcD	map00630	Glyoxylate and dicarboxylate metabolism
gene3626	uroporphyrinogen-III synthase	9	26	K01719	hemD	map00860	Porphyrin and chlorophyll metabolism
gene3629	asparagine synthase	3	1	K01953	asnB	map00250	Alanine, aspartate and glutamate metabolism
gene3680	initiation factor 2B subunit alpha	53	25	K08963	mtnA	map00270	Cysteine and methionine metabolism
gene3756	Zn-dependent hydrolase	2	4	K06016	pydC	map00240	Pyrimidine metabolism
gene3757	urocanate hydratase	4	12	K01712	hutU	map00340	Histidine metabolism
gene3819	phosphoenolpyruvate carboxylase	208	66	K01595	ppc	map00680;map00720;map00620;map00710	Methane metabolism;Carbon fixation pathways in prokaryotes;Pyruvate metabolism;Carbon fixation in photosynthetic organisms
gene3920	PTS lactose transporter subunit IIC	3	7	K02770	fruA	map02060;map00051	Phosphotransferase system (PTS);Fructose and mannose metabolism
gene3921	phosphofructokinase	2	5	K00882	fruK	map02060;map00051	Phosphotransferase system (PTS);Fructose and mannose metabolism
gene3952	hypothetical protein	3	1	K14340	------	map00571	Lipoarabinomannan (LAM) biosynthesis
gene3958	transcriptional regulator	23	7	K05825	------	map00300	Lysine biosynthesis
gene4014	cyclohex-1-ene-1-carboxylate:CoA ligase	0	0	K04116	aliA	map00362	Benzoate degradation
gene4019	glycerol-3-phosphate dehydrogenase	6	25	K00111	glpA	map00564	Glycerophospholipid metabolism
gene4145	NADH-quinone oxidoreductase subunit M	58	133	K00342	nuoM	map00190	Oxidative phosphorylation
gene4151	NADH-quinone oxidoreductase subunit G	38	91	K00336	nuoG	map00190	Oxidative phosphorylation
gene4161	D-alanyl-D-alanine carboxypeptidase	367	94	K07258	dacC	map00550	Peptidoglycan biosynthesis
gene4190	hypothetical protein	4	2	K03365	------	map00240;map00330	Pyrimidine metabolism;Arginine and proline metabolism
gene4387	CoA ester lyase	13	5	K08691	mcl	map00680;map00630;map00660;map00720	Methane metabolism;Glyoxylate and dicarboxylate metabolism;C5-Branched dibasic acid metabolism;Carbon fixation pathways in prokaryotes
gene4388	CoA ester lyase	11	5	K01644	citE	map02020	Two-component system
gene4418	precorrin-6A reductase	3	1	K05895	cobK-cbiJ	map00860	Porphyrin and chlorophyll metabolism
gene4431	ABC transporter permease	0	0	K05846	opuBD	map02010	ABC transporters
gene4462	hypothetical protein	1	3	K07695	devR	map02020	Two-component system
gene4485	phenylacetate-CoA oxygenase subunit PaaB	21	10	K02610	paaB	map00360	Phenylalanine metabolism

gene4625	diaminopimelate decarboxylase	9	4	K01586	lysA	map00300	Lysine biosynthesis
gene4665	hypothetical protein	28	13	K08372	pepD	map02020	Two-component system
gene4666	putative serine protease	271	101	K08372	pepD	map02020	Two-component system
gene4874	Carbonic anhydrase	0	1	K01673	cynT	map00910	Nitrogen metabolism
gene4902	glycerophosphodiester phosphodiesterase	0	1	K01126	glpQ	map00564	Glycerophospholipid metabolism
gene5020	pyrroline-5-carboxylate reductase	75	31	K00286	proC	map00330	Arginine and proline metabolism
gene5030	pyridine nucleotide-disulfide oxidoreductase	2	1	K00529	hcaD	map00360;map00071	Phenylalanine metabolism;Fatty acid degradation
gene5032	3-oxoacyl-ACP synthase	15	2	K09458	fabF	map00780;map00061	Biotin metabolism;Fatty acid biosynthesis
gene5034	3-oxoacyl-ACP synthase	5	1	K09458	fabF	map00780;map00061	Biotin metabolism;Fatty acid biosynthesis
gene5037	3-oxoacyl-ACP reductase	8	1	K00059	fabG	map00780;map00061;map00333	Biotin metabolism;Fatty acid biosynthesis;Prodigiosin biosynthesis
gene5038	short-chain dehydrogenase	3	1	K12420	actIII	map00253;map01056	Tetracycline biosynthesis;Biosynthesis of type II polyketide backbone
gene5066	phosphate ABC transporter ATP-binding protein	3	1	K02036	pstB	map02010	ABC transporters
gene5067	phosphate ABC transporter, permease protein PstA	2	0	K02038	pstA	map02010	ABC transporters
gene5068	phosphate ABC transporter permease subunit PstC	4	1	K02037	pstC	map02010	ABC transporters
gene5069	phosphate ABC transporter substrate-binding protein PstS	11	1	K02040	pstS	map05152;map02010;map02020	Tuberculosis;ABC transporters;Two-component system
gene5106	multidrug ABC transporter ATP-binding protein	43	16	K18887	efrA	map02010	ABC transporters
gene5107	multidrug ABC transporter ATP-binding protein	28	13	K18888	efrB	map02010	ABC transporters
gene5116	bifunctional metallophosphatase/5'-nucleotidase	12	3	K01081	------	map00240;map00230;map00760	Pyrimidine metabolism;Purine metabolism;Nicotinate and nicotinamide metabolism
gene5206	threonine synthase	145	50	K01733	thrC	map00750;map00260	Vitamin B6 metabolism;Glycine, serine and threonine metabolism
gene5221	glutamate transporter glutamate-binding protein	21	6	K10005	gluB	map02010	ABC transporters
gene5266	phosphoserine aminotransferase	230	77	K00831	serC	map00680;map00270;map00750;map00260	Methane metabolism;Cysteine and methionine metabolism;Vitamin B6 metabolism;Glycine, serine and threonine metabolism
gene5276	acetyl-CoA carboxylase subunit alpha	2	1	K01965	pccA	map00630;map00280;map00640	Glyoxylate and dicarboxylate metabolism;Valine, leucine and isoleucine degradation;Propanoate metabolism
gene5314	cholesterol oxidase	6	2	K03333	choD	map00984	Steroid degradation
gene5381	hypothetical protein	3	1	K16937	doxD	map00920	Sulfur metabolism
gene5443	glycine betaine aldehyde dehydrogenase	4	12	K00130	betB	map00260	Glycine, serine and threonine metabolism
gene5445	glycine/betaine ABC transporter ATP-binding protein	1	4	K02000	proV	map02010	ABC transporters
gene5447	ABC transporter permease	1	3	K02001	proW	map02010	ABC transporters
gene5448	glycine/betaine ABC transporter substrate-binding protein	2	7	K02002	proX	map02010	ABC transporters
gene5460	D-alanyl-D-alanine carboxypeptidase	35	19	K07258	dacC	map00550	Peptidoglycan biosynthesis
gene5518	adenosine deaminase	9	4	K01488	add	map05340;map00230	Primary immunodeficiency;Purine metabolism
gene5541	purine-nucleoside phosphorylase	26	14	K03783	punA	map00240;map00230;map00760	Pyrimidine metabolism;Purine metabolism;Nicotinate and nicotinamide metabolism
gene5572	cystathionine gamma-synthase	52	27	K01739	metB	map00450;map00920;map00270	Selenocompound metabolism;Sulfur metabolism;Cysteine and methionine metabolism
gene5576	threonine dehydratase	7	4	K01754	ilvA	map00260;map00290	Glycine, serine and threonine metabolism;Valine, leucine and isoleucine biosynthesis
gene5591	phosphoenolpyruvate carboxykinase	26	69	K01596	pckA	map04920;map04964;map00620;map04068;map00010;map04922;map04910;map03320;map00020;map04151;map04152;map04931	Citrate cycle (TCA cycle);PI3K-Akt signaling pathway;AMPK signaling pathway;Insulin resistance
gene5638	catalase	10	45	K03781	katE	map00630;map04016;map04011;map05208;map04068;map05014;map04146;map04213;map04212;map04211;map05022;map00380	Glyoxylate and dicarboxylate metabolism;MAPK signaling pathway - plant;MAPK signaling pathway - yeast;Chemical carcinogenesis - reactive oxygen species;FoxO signaling pathway;Amyotrophic lateral sclerosis;Peroxisome;Longevity regulating pathway - multiple species;Longevity regulating pathway - worm;Longevity regulating pathway;Pathways of neurodegeneration - multiple diseases;Tryptophan metabolism
gene5648	fructose 1,6-bisphosphatase	36	135	K02446	glpX	map00680;map00010;map00030;map00051;map00710	Methane metabolism;Glycolysis / Gluconeogenesis;Pentose phosphate pathway;Fructose and mannose metabolism;Carbon fixation in photosynthetic organisms
gene5675	peptide ABC transporter substrate-binding protein	1	2	K15580	oppA	map02024;map02010;map01501	Quorum sensing;ABC transporters;beta-Lactam resistance
gene5677	peptide ABC transporter permease	0	1	K15582	oppC	map02024;map02010;map01501	Quorum sensing;ABC transporters;beta-Lactam resistance
gene5678	Oligopeptide transport ATP-binding protein OppD	0	1	K15583	oppD	map02024;map02010;map01501	Quorum sensing;ABC transporters;beta-Lactam resistance

gene5679	peptide ABC transporter ATP-binding protein	0	1	K10823	oppF	map02024;map02010;map01501	Quorum sensing;ABC transporters;beta-Lactam resistance
gene5790	catalase	103	374	K03781	katE	map00630;map04016;map04011;map05208;map04068;map05014;map04146;map04213;map04212;map04211;map05022;map00380	Glyoxylate and dicarboxylate metabolism;MAPK signaling pathway - plant;MAPK signaling pathway - yeast;Chemical carcinogenesis - reactive oxygen species;FoxO signaling pathway;Amyotrophic lateral sclerosis;Peroxisome;Longevity regulating pathway - multiple species;Longevity regulating pathway - worm;Longevity regulating pathway;Pathways of neurodegeneration - multiple diseases;Tryptophan metabolism
gene5815	hypothetical protein	10	5	K17329	dasA	map02010	ABC transporters
gene5817	sugar transporter integral membrane protein	4	2	K17330	dasB	map02010	ABC transporters
gene5900	diaminopimelate decarboxylase	67	24	K01586	lysA	map00300	Lysine biosynthesis
gene5913	serine hydroxymethyltransferase	6	18	K00600	glyA	map00630;map00460;map00680;map00260;map01523;map00670	Glyoxylate and dicarboxylate metabolism;Cyanoamino acid metabolism;Methane metabolism;Glycine, serine and threonine metabolism;Antifolate resistance;One carbon pool by folate
gene5971	isobutyryl-CoA mutase, chain A	15	34	K01848	mcmA1	map00630;map00720;map00280;map00640	Glyoxylate and dicarboxylate metabolism;Carbon fixation pathways in prokaryotes;Valine, leucine and isoleucine degradation;Propanoate metabolism
gene6064	phospholipase C, phosphocholine-specific	3	2	K01114	plc	map00565;map00564;map02024;map04919;map00562	Ether lipid metabolism;Glycerophospholipid metabolism;Quorum sensing;Thyroid hormone signaling pathway;Inositol phosphate metabolism
gene6078	3-phosphoglycerate dehydrogenase	305	149	K00058	serA	map00680;map00270;map00260	Methane metabolism;Cysteine and methionine metabolism;Glycine, serine and threonine metabolism
gene6088	3-isopropylmalate dehydrogenase	385	120	K00052	leuB	map00660;map00290	C5-Branched dibasic acid metabolism;Valine, leucine and isoleucine biosynthesis
gene6123	3-isopropylmalate dehydratase large subunit	226	69	K01703	leuC	map00660;map00966;map00290	C5-Branched dibasic acid metabolism;Glucosinolate biosynthesis;Valine, leucine and isoleucine biosynthesis
gene6124	3-isopropylmalate dehydratase small subunit	139	45	K01704	leuD	map00660;map00290	C5-Branched dibasic acid metabolism;Valine, leucine and isoleucine biosynthesis
gene6148	acylphosphatase	7	16	K01512	acyP	map00627;map00620	Aminobenzoate degradation;Pyruvate metabolism
gene6164	-	3	1	K00990	glnD	map02020	Two-component system
gene6165	signal recognition particle protein	80	194	K03106	ffh	map02024;map03070;map03060	Quorum sensing;Bacterial secretion system;Protein export
gene6193	DNA-directed RNA polymerase sigma-70 factor	7	25	K02405	fliA	map02026;map02025;map02040;map02020;map05111	Biofilm formation - Escherichia coli;Biofilm formation - Pseudomonas aeruginosa;Flagellar assembly;Two-component system;Biofilm formation - Vibrio cholerae
gene6203	ABC transporter integral membrane protein	3	8	K02063	thiP	map02010	ABC transporters
gene6215	gamma-aminobutyraldehyde dehydrogenase	76	303	K00130	betB	map00260	Glycine, serine and threonine metabolism
gene6230	aspartate aminotransferase family protein	107	28	K07250	gabT	map00310;map00640;map00650;map00250;map00280;map00410	Lysine degradation;Propanoate metabolism;Butanoate metabolism;Alanine, aspartate and glutamate metabolism;Valine, leucine and isoleucine degradation;beta-Alanine metabolism
gene6240	4-hydroxy-3-methylbut-2-en-1-yl diphosphate synthase	149	348	K03526	gcpE	map00900	Terpenoid backbone biosynthesis
gene6305	CDP-diacylglycerol--glycerol-3-phosphate 3-phosphatidyltransferase	31	109	K00995	pgsA	map00564	Glycerophospholipid metabolism
gene6306	competence-damage inducible protein	39	127	K03743	pncC	map00760	Nicotinate and nicotinamide metabolism
gene6321	sec-independent translocase	14	81	K03117	tatB	map03070;map03060	Bacterial secretion system;Protein export
gene6333	FAD-binding monooxygenase	6	15	K05712	mhpA	map00360	Phenylalanine metabolism
gene6351	kinase	20	46	K01139	spoT	map00230	Purine metabolism
gene6402	hypothetical protein	4	81	K01011	sseA	map00270;map04122;map00920	Cysteine and methionine metabolism;Sulfur relay system;Sulfur metabolism
gene6416	deoxyuridine 5'-triphosphate nucleotidohydrolase	46	122	K01520	dut	map00240;map00983	Pyrimidine metabolism;Drug metabolism - other enzymes
gene6419	potassium-transporting ATPase subunit A	7	3	K01546	kdpA	map02020	Two-component system
gene6420	potassium-transporting ATPase subunit B	3	2	K01547	kdpB	map02020	Two-component system
gene6468	putative hydrolase	424	1244	K01091	gph	map00630	Glyoxylate and dicarboxylate metabolism
gene6473	methylmalonate-semialdehyde dehydrogenase (acylating)	0	1	K00140	mmsA	map00280;map00640;map00410;map00562	Valine, leucine and isoleucine degradation;Propanoate metabolism;beta-Alanine metabolism;Inositol phosphate metabolism
gene6476	hypothetical protein	0	2	K00020	mmsB	map00280	Valine, leucine and isoleucine degradation
gene6477	enoyl-CoA hydratase	1	3	K01692	paaF	map00310;map00640;map00281;map00650;map00627;map00903;map00930;map00380;map00362;map00360;map00071;map00280;map00410	Lysine degradation;Propanoate metabolism;Geraniol degradation;Butanoate metabolism;Aminobenzoate degradation;Limonene and pinene degradation;Caprolactam degradation;Tryptophan metabolism;Benzoate degradation;Phenylalanine metabolism;Fatty acid degradation;Valine, leucine and isoleucine degradation;beta-Alanine metabolism
gene6478	aspartate 1-decarboxylase	2	15	K01579	panD	map00770;map00410	Pantothenate and CoA biosynthesis;beta-Alanine metabolism
gene6479	MbtH family protein	3	42	K05375	mbtH	map00261	Monobactam biosynthesis
gene6497	3-oxoacyl-ACP synthase	2	6	K00648	fabH	map00061	Fatty acid biosynthesis

gene6498	Glutamate dehydrogenase	2	5	K00261	gdhA	map04964;map00220;map00250;map00910;map04217	Proximal tubule bicarbonate reclamation;Arginine biosynthesis;Alanine, aspartate and glutamate metabolism;Nitrogen metabolism;Necroptosis
gene6499	hypothetical protein	1	3	K12253	aruI	map00330	Arginine and proline metabolism
gene6501	hypothetical protein	1	6	K09458	fabF	map00780;map00061	Biotin metabolism;Fatty acid biosynthesis
gene6502	hypothetical protein	1	8	K18661	matB	map00280	Valine, leucine and isoleucine degradation
gene6503	hypothetical protein	2	16	K16025	asm14	map01052;map01051	Type I polyketide structures;Biosynthesis of ansamycins
gene6524	ABC transporter substrate-binding protein	51	115	K10543	xylF	map02010	ABC transporters
gene6526	ABC transporter permease	9	21	K10544	xylH	map02010	ABC transporters
gene6537	hypothetical protein	4	12	K00141	xylC	map00627;map00622;map00623	Aminobenzoate degradation;Xylene degradation;Toluene degradation
gene6558	ABC transporter ATPase	3	7	K09814	hrtA	map02010	ABC transporters
gene6559	ABC transporter substrate-binding protein	3	11	K09813	hrtB	map02010	ABC transporters
gene6571	peptidase M15	1	3	K08641	vanX	map01502;map02020	Vancomycin resistance;Two-component system
gene6572	malto-oligosyltrehalose trehalohydrolase	2	5	K01236	treZ	map00500	Starch and sucrose metabolism
gene6578	glycogen debranching enzyme	10	38	K01214	treX	map00500	Starch and sucrose metabolism
gene6633	(2Fe-2S)-binding protein	3	10	K13483	yagT	map00230	Purine metabolism
gene6635	xanthine dehydrogenase	1	5	K11177	yagR	map00230	Purine metabolism
gene6637	phospholipase C, phosphocholine-specific	7	17	K01114	plc	map00565;map00564;map02024;map04919;map00562	Ether lipid metabolism;Glycerophospholipid metabolism;Quorum sensing;Thyroid hormone signaling pathway;Inositol phosphate metabolism
gene6668	ribosome small subunit-dependent GTPase A	73	36	K06949	rsgA	map00730	Thiamine metabolism
gene6672	pyruvate dehydrogenase	2	6	K00156	poxB	map00620	Pyruvate metabolism
gene6719	glyoxylate carboligase	0	1	K01608	gcl	map00630	Glyoxylate and dicarboxylate metabolism
gene6720	2-hydroxy-3-oxopropionate reductase	1	3	K00042	garR	map00630	Glyoxylate and dicarboxylate metabolism
gene6721	oxidoreductase	1	3	K01816	hyi	map00630	Glyoxylate and dicarboxylate metabolism
gene6730	chitosanase	14	61	K01233	csn	map00520	Amino sugar and nucleotide sugar metabolism
gene6743	allantoinase	4	10	K01466	allB	map00230	Purine metabolism
gene6744	allantoicase	4	10	K01477	alc	map00230	Purine metabolism
gene6937	acetyltransferase	15	38	K00657	speG	map00330;map04216	Arginine and proline metabolism;Ferroptosis
gene6939	lanthionine synthetase C-like protein	16	40	K20484	nisC	map02024;map02020	Quorum sensing;Two-component system
gene6940	lantibiotic dehydratase	11	28	K20483	nisB	map02024;map02020	Quorum sensing;Two-component system
gene6991	dihydropyrimidinase	71	168	K01464	dht	map00240;map00983;map00770;map00410	Pyrimidine metabolism;Drug metabolism - other enzymes;Pantothenate and CoA biosynthesis;beta-Alanine metabolism
gene7010	CDP-diacylglycerol--serine O-phosphatidyltransferase	67	221	K17103	pssA	map00564;map00260	Glycerophospholipid metabolism;Glycine, serine and threonine metabolism
gene7011	phosphatidylserine decarboxylase	80	200	K01613	psd	map00564	Glycerophospholipid metabolism
gene7042	arginase	66	180	K01476	rocF	map00220;map00330;map05146	Arginine biosynthesis;Arginine and proline metabolism;Amoebiasis
gene7058	4a-hydroxytetrahydrobiopterin dehydratase	16	57	K01724	phhB	map00790	Folate biosynthesis
gene7067	long-chain acyl-CoA synthetase	2	8	K01897	fadD	map04714;map02024;map03320;map04920;map00061;map00071;map04146;map04216	Thermogenesis;Quorum sensing;PPAR signaling pathway;Adipocytokine signaling pathway;Fatty acid biosynthesis;Fatty acid degradation;Peroxisome;Ferroptosis
gene7070	neuraminidase	0	1	K01186	------	map00600;map00511;map04142	Sphingolipid metabolism;Other glycan degradation;Lysosome
gene7081	acetolactate synthase	8	35	K01652	ilvB	map00660;map00290;map00770;map00650	C5-Branched dibasic acid metabolism;Valine, leucine and isoleucine biosynthesis;Pantothenate and CoA biosynthesis;Butanoate metabolism
gene7133	haloacid dehalogenase	24	79	K01091	gph	map00630	Glyoxylate and dicarboxylate metabolism
gene7152	phosphopantothenoyl cysteine decarboxylase	2	6	K13038	coaBC	map00770	Pantothenate and CoA biosynthesis
gene7153	hypothetical protein	3	10	K00320	mer	map00680	Methane metabolism
gene7155	O-methyltransferase	4	17	K20421	ncsB1	map01059	Biosynthesis of enediyne antibiotics
gene7202	oxidoreductase	0	0	K13327	spnN	map00523	Polyketide sugar unit biosynthesis
gene7252	6-pyruvoyl tetrahydrobiopterin synthase	1	72	K01737	queD	map00790	Folate biosynthesis
gene7254	7-carboxy-7-deazaguanine synthase QueE	1	55	K10026	queE	map00790	Folate biosynthesis
gene7255	GTP cyclohydrolase	2	76	K01495	folE	map00790	Folate biosynthesis
gene7256	guanosine monophosphate reductase	1	32	K00088	guaB	map00983;map00230	Drug metabolism - other enzymes;Purine metabolism
gene7257	adenylosuccinate lyase	2	36	K01756	purB	map00250;map00230	Alanine, aspartate and glutamate metabolism;Purine metabolism
gene7258	adenylosuccinate synthetase	1	22	K01939	purA	map00250;map00230	Alanine, aspartate and glutamate metabolism;Purine metabolism
gene7261	7-cyano-7-deazaguanine synthase	2	70	K06920	queC	map00790	Folate biosynthesis

gene7273	deoxycytidine triphosphate deaminase	7	16	K01494	dcd	map00240	Pyrimidine metabolism
gene7316	two-component sensor histidine kinase	20	71	K07654	mtrB	map02020	Two-component system
gene7363	hypothetical protein	3	9	K00273	aao	map04146;map00311;map00330;map00260	Peroxisome;Penicillin and cephalosporin biosynthesis;Arginine and proline metabolism;Glycine, serine and threonine metabolism
gene7386	squalene synthase HpnD	36	99	K02291	crtB	map00906	Carotenoid biosynthesis
gene7392	aspartate aminotransferase family protein	24	64	K00819	rocD	map00330	Arginine and proline metabolism
gene7438	hypothetical protein	3	25	K01915	glnA	map00630;map00220;map00910;map02020;map00250;map04727;map04724;map04217	Glyoxylate and dicarboxylate metabolism;Arginine biosynthesis;Nitrogen metabolism;Two-component system;Alanine, aspartate and glutamate metabolism;GABAergic synapse;Glutamatergic synapse;Necroptosis
gene7442	NADP-dependent succinic semialdehyde dehydrogenase	1	9	K00135	gabD	map00250;map00310;map00760;map00350;map00650	Alanine, aspartate and glutamate metabolism;Lysine degradation;Nicotinate and nicotinamide metabolism;Tyrosine metabolism;Butanoate metabolism
gene7446	hypothetical protein	3	1	K14379	------	map04380;map05323;map04142;map00740	Osteoclast differentiation;Rheumatoid arthritis;Lysosome;Riboflavin metabolism
gene7447	enoyl-CoA hydratase	5	0	K01692	paaF	map00310;map00640;map00281;map00650;map00627;map00903;map00930;map00380;map00362;map00360;map00071;map00280;map00410	Lysine degradation;Propanoate metabolism;Geraniol degradation;Butanoate metabolism;Aminobenzoate degradation;Limonene and pinene degradation;Caprolactam degradation;Tryptophan metabolism;Benzoate degradation;Phenylalanine metabolism;Fatty acid degradation;Valine, leucine and isoleucine degradation;beta-Alanine metabolism
gene7461	hypothetical protein	2	25	K01808	rpiB	map00030;map00051;map00710	Pentose phosphate pathway;Fructose and mannose metabolism;Carbon fixation in photosynthetic organisms
gene7490	phosphatidyl inositol-specific phospholipase C	5	18	K01771	plc	map00562	Inositol phosphate metabolism
gene7491	dimethylhistidine N-methyltransferase	11	56	K18911	egtD	map00340	Histidine metabolism
gene7492	class II glutamine amidotransferase	5	20	K07008	egtC	map00340	Histidine metabolism
gene7493	ergothioneine biosynthesis protein EgtB	9	34	K18912	egtB	map00340	Histidine metabolism
gene7494	ergothioneine biosynthesis glutamate--cysteine ligase EgtA	21	95	K01919	gshA	map00270;map00480	Cysteine and methionine metabolism;Glutathione metabolism
gene7495	aminotransferase AlaT	6	47	K14260	alaA	map00220;map00250;map00290	Arginine biosynthesis;Alanine, aspartate and glutamate metabolism;Valine, leucine and isoleucine biosynthesis
gene7517	carbohydrate kinase	1	4	K22935	psk	map00040	Pentose and glucuronate interconversions
gene7519	D-isomer specific 2-hydroxyacid dehydrogenase NAD-binding protein	2	7	K00058	serA	map00680;map00270;map00260	Methane metabolism;Cysteine and methionine metabolism;Glycine, serine and threonine metabolism
gene7553	acyl-CoA thioesterase II	9	22	K10805	tesB	map01040	Biosynthesis of unsaturated fatty acids
gene7561	glycosidase	1	5	K01176	amyA	map04972;map04973;map04970;map00500	Pancreatic secretion;Carbohydrate digestion and absorption;Salivary secretion;Starch and sucrose metabolism
gene7576	ABC transporter	4	1	K15555	ssuB	map02010;map00920	ABC transporters;Sulfur metabolism
gene7577	ABC transporter permease	3	1	K15554	ssuC	map02010;map00920	ABC transporters;Sulfur metabolism
gene7609	solute-binding protein	7	23	K10543	xylF	map02010	ABC transporters
gene7613	hypothetical protein	3	10	K05349	bglX	map00460;map00940;map00500	Cyanoamino acid metabolism;Phenylpropanoid biosynthesis;Starch and sucrose metabolism
gene7638	alpha-glucosidase	1	8	K01187	malZ	map00500;map00052	Starch and sucrose metabolism;Galactose metabolism
gene7645	short-chain dehydrogenase	2	34	K03366	butA	map00650	Butanoate metabolism
gene7648	aspartate aminotransferase family protein	2	34	K00822	------	map00280;map00640;map00410	Valine, leucine and isoleucine degradation;Propanoate metabolism;beta-Alanine metabolism
gene7664	ribonucleoside hydrolase	1	2	K01239	iunH	map00230;map00760	Purine metabolism;Nicotinate and nicotinamide metabolism
gene7681	antibiotic ABC transporter ATP-binding protein	16	3	K18230	tylC	map02010	ABC transporters
gene7685	pyridoxal-5'-phosphate-dependent protein subunit beta	1	1	K22847	cysK2	map00270	Cysteine and methionine metabolism
gene7690	membrane protein	3	11	K03118	tatC	map03070;map03060	Bacterial secretion system;Protein export
gene7691	protein translocase TatA	2	14	K03116	tatA	map03070;map03060	Bacterial secretion system;Protein export
gene7707	two-component sensor histidine kinase	14	33	K07654	mtrB	map02020	Two-component system
gene7710	beta-N-acetylglucosaminidase	1	3	K01197	hya	map04142;map00531	Lysosome;Glycosaminoglycan degradation
gene7725	aldehyde dehydrogenase	13	36	K00138	aldB	map00010;map00620	Glycolysis / Gluconeogenesis;Pyruvate metabolism
gene7730	glycoside hydrolase	1	5	K01183	------	map00520	Amino sugar and nucleotide sugar metabolism
gene7741	glycosyl transferase	22	50	K00721	------	map00510	N-Glycan biosynthesis
gene7745	transmembrane efflux protein	9	23	K06902	------	map04138	Autophagy - yeast
gene7746	phosphodiesterase	3	8	K01126	glpQ	map00564	Glycerophospholipid metabolism
gene7756	D-alanyl-D-alanine carboxypeptidase	0	1	K07258	dacC	map00550	Peptidoglycan biosynthesis
gene7821	nicotinate phosphoribosyltransferase	0	1	K00763	pncB	map00760	Nicotinate and nicotinamide metabolism

gene7826	glycerophosphodiester phosphodiesterase	0	2	K01126	glpQ	map00564	Glycerophospholipid metabolism
gene7862	dethiobiotin synthase	0	2	K01935	bioD	map00780	Biotin metabolism
gene7886	Lipoprotein releasing system ATP-binding protein LolD	37	192	K09810	lolD	map02010	ABC transporters
gene7930	6-phosphofructokinase	95	312	K21071	pfk	map00680;map00010;map00030;map00051;map00052	Methane metabolism;Glycolysis / Gluconeogenesis;Pentose phosphate pathway;Fructose and mannose metabolism;Galactose metabolism
gene7955	glyoxylate carboligase	0	1	K01608	gcl	map00630	Glyoxylate and dicarboxylate metabolism
gene7971	cytochrome C3-like	2	9	K06282	hyaA	map00633;map02020	Nitrotoluene degradation;Two-component system
gene7972	cytochrome C3-like	1	4	K06281	hyaB	map00633;map02020	Nitrotoluene degradation;Two-component system
gene8046	3-oxoacyl-ACP reductase	0	2	K00038	------	map00140	Steroid hormone biosynthesis
gene8049	hypothetical protein	0	3	K16048	hsaB	map00984	Steroid degradation
gene8050	long-chain-fatty-acid--CoA ligase	0	1	K18687	fadD3	map00984	Steroid degradation
gene8054	enoyl-CoA hydratase	0	5	K01692	paaF	map00310;map00640;map00281;map00650;map00627;map00903;map00930;map00380;map00362;map00360;map00071;map00280;map00410	Lysine degradation;Propanoate metabolism;Geraniol degradation;Butanoate metabolism;Aminobenzoate degradation;Limonene and pinene degradation;Caprolactam degradation;Tryptophan metabolism;Benzoate degradation;Phenylalanine metabolism;Fatty acid degradation;Valine, leucine and isoleucine degradation;beta-Alanine metabolism
gene8056	short chain dehydrogenase	0	4	K00059	fabG	map00780;map00061;map00333	Biotin metabolism;Fatty acid biosynthesis;Prodigiosin biosynthesis
gene8058	acetyl-CoA acetyltransferase	1	52	K00626	atoB	map00630;map00310;map00640;map00650;map00720;map00620;map00900;map00362;map02020;map00071;map00280;map04975;map00380	Glyoxylate and dicarboxylate metabolism;Lysine degradation;Propanoate metabolism;Butanoate metabolism;Carbon fixation pathways in prokaryotes;Pyruvate metabolism;Terpenoid backbone biosynthesis;Benzoate degradation;Two-component system;Fatty acid degradation;Valine, leucine and isoleucine degradation;Fat digestion and absorption;Tryptophan metabolism
gene8062	CoA-transferase	0	3	K01039	gctA	map00643;map00650	Styrene degradation;Butanoate metabolism
gene8063	enoyl-CoA hydratase	0	2	K01692	paaF	map00310;map00640;map00281;map00650;map00627;map00903;map00930;map00380;map00362;map00360;map00071;map00280;map00410	Lysine degradation;Propanoate metabolism;Geraniol degradation;Butanoate metabolism;Aminobenzoate degradation;Limonene and pinene degradation;Caprolactam degradation;Tryptophan metabolism;Benzoate degradation;Phenylalanine metabolism;Fatty acid degradation;Valine, leucine and isoleucine degradation;beta-Alanine metabolism
gene8066	2-keto-4-pentenoate hydratase	0	0	K02554	mhpD	map00362;map00360;map00622;map00621	Benzoate degradation;Phenylalanine metabolism;Xylene degradation;Dioxin degradation
gene8067	acetaldehyde dehydrogenase	0	1	K04073	mhpF	map00650;map00622;map00620;map00621;map00362;map00360	Butanoate metabolism;Xylene degradation;Pyruvate metabolism;Dioxin degradation;Benzoate degradation;Phenylalanine metabolism
gene8069	dioxygenase Rieske iron-sulfur component	0	2	K15982	kshA	map00984	Steroid degradation
gene8071	3-ketosteroid-9-alpha-hydroxylase reductase subunit	0	1	K15983	kshB	map00984	Steroid degradation
gene8073	monooxygenase	0	1	K16047	hsaA	map00984	Steroid degradation
gene8074	2-hydroxy-6-ketonona-2,4-dienedioic acid hydrolase	0	1	K16050	hsaD	map00984	Steroid degradation
gene8075	glyoxalase/bleomycin resistance protein/dioxygenase	0	1	K16049	hsaC	map00984	Steroid degradation
gene8078	3-oxosteroid 1-dehydrogenase	0	0	K05898	kstD	map00984	Steroid degradation
gene8086	acid phosphatase	0	1	K21302	sapM	map05152;map00562	Tuberculosis;Inositol phosphate metabolism
gene8088	CoA transferase	0	2	K18289	ict-P	map00660	C5-Branched dibasic acid metabolism
gene8100	hypothetical protein	0	0	K00812	aspB	map00220;map00270;map00330;map00950;map00250;map00360;map00350;map00960;map00400;map00401	Arginine biosynthesis;Cysteine and methionine metabolism;Arginine and proline metabolism;Isoquinoline alkaloid biosynthesis;Alanine, aspartate and glutamate metabolism;Phenylalanine metabolism;Tyrosine metabolism;Tropane, piperidine and pyridine alkaloid biosynthesis;Phenylalanine, tyrosine and tryptophan biosynthesis;Novobiocin biosynthesis
gene8101	hypothetical protein	0	2	K16163	------	map00350	Tyrosine metabolism
gene8104	acyl-CoA dehydrogenase	0	1	K00249	acd	map00280;map03320;map00071	Valine, leucine and isoleucine degradation;PPAR signaling pathway;Fatty acid degradation
gene8105	hypothetical protein	0	1	K11731	atuD	map00281	Geraniol degradation
gene8106	hypothetical protein	0	0	K06133	acpT	map00770	Pantothenate and CoA biosynthesis
gene8114	alcohol dehydrogenase	0	2	K13953	adhP	map00071;map00980;map00626;map00625;map00620;map00010;map00830;map00350;map00982	Fatty acid degradation;Metabolism of xenobiotics by cytochrome P450;Naphthalene degradation;Chloroalkane and chloroalkene degradation;Pyruvate metabolism;Glycolysis / Gluconeogenesis;Retinol metabolism;Tyrosine metabolism;Drug metabolism - cytochrome P450
gene8119	protein translocase TatA	60	191	K03116	tatA	map03070;map03060	Bacterial secretion system;Protein export
gene8133	lysine/ornithine decarboxylase	4	10	K01586	lysA	map00300	Lysine biosynthesis
gene8135	cysteine desulfurase	2	5	K11717	sufS	map00450	Selenocompound metabolism
gene8158	squalene-hopene cyclase	7	36	K06045	shc	map00909	Sesquiterpenoid and triterpenoid biosynthesis
gene8202	hypothetical protein	4	38	K01470	------	map00330	Arginine and proline metabolism
gene8203	hypothetical protein	4	36	K17828	pyrDI	map00240	Pyrimidine metabolism
gene8206	ornithine--oxo-acid aminotransferase	5	35	K00821	argD	map00220;map00300	Arginine biosynthesis;Lysine biosynthesis
gene8208	hypothetical protein	5	44	K02372	fabZ	map00780;map00061	Biotin metabolism;Fatty acid biosynthesis
gene8209	hypothetical protein	1	6	K09458	fabF	map00780;map00061	Biotin metabolism;Fatty acid biosynthesis
gene8219	6-aminohexanoate hydrolase	1	2	K01426	amiE	map00627;map00360;map00330;map00380;map00643	Aminobenzoate degradation;Phenylalanine metabolism;Arginine and proline metabolism;Tryptophan metabolism;Styrene degradation
gene8249	methionine synthase	0	4	K00548	metH	map00450;map00670;map00270	Selenocompound metabolism;One carbon pool by folate;Cysteine and methionine metabolism

gene8250	carbohydrate kinase family protein	0	1	K00856	------	map00230	Purine metabolism
gene8251	methionine adenosyltransferase	0	2	K00789	metK	map00270	Cysteine and methionine metabolism
gene8256	2-isopropylmalate synthase	0	11	K01649	leuA	map00620;map00290	Pyruvate metabolism;Valine, leucine and isoleucine biosynthesis
gene8257	hypothetical protein	0	1	K01649	leuA	map00620;map00290	Pyruvate metabolism;Valine, leucine and isoleucine biosynthesis
gene8258	isopropylmalate isomerase	0	2	K01703	leuC	map00660;map00966;map00290	C5-Branched dibasic acid metabolism;Glucosinolate biosynthesis;Valine, leucine and isoleucine biosynthesis
gene8259	3-isopropylmalate dehydrogenase	0	1	K00052	leuB	map00660;map00290	C5-Branched dibasic acid metabolism;Valine, leucine and isoleucine biosynthesis
gene8260	branched chain amino acid aminotransferase	0	1	K00826	ilvE	map00270;map00770;map00280;map00966;map00290	Cysteine and methionine metabolism;Pantothenate and CoA biosynthesis;Valine, leucine and isoleucine degradation;Glucosinolate biosynthesis;Valine, leucine and isoleucine biosynthesis
gene8295	limonene 1,2-monooxygenase	1	0	K14733	limB	map00903	Limonene and pinene degradation
gene8311	cyclic nucleotide-regulated FAD-dependent pyridine nucleotide-disulfide oxidoreductase	1	1	K00384	trxB	map00450	Selenocompound metabolism
gene8355	NAD(P)H quinone dehydrogenase	1	0	K00355	------	map05225;map00130;map05208;map05418;map05200	Hepatocellular carcinoma;Ubiquinone and other terpenoid-quinone biosynthesis;Chemical carcinogenesis - reactive oxygen species;Fluid shear stress and atherosclerosis;Pathways in cancer
gene8357	dioxygenase	1	0	K00459	ncd2	map00910	Nitrogen metabolism
gene8387	chitinase	0	0	K01183	------	map00520	Amino sugar and nucleotide sugar metabolism
gene8395	xylulokinase	0	0	K00854	xylB	map00040	Pentose and glucuronate interconversions
gene8417	dimethylmenaquinone methyltransferase	2	0	K10218	ligK	map00362;map00660	Benzoate degradation;C5-Branched dibasic acid metabolism
gene8418	hypothetical protein	2	0	K22552	mmcO	map00860	Porphyrin and chlorophyll metabolism
gene8425	serine/threonine protein kinase	19	9	K00613	------	map00330;map00260	Arginine and proline metabolism;Glycine, serine and threonine metabolism
gene8426	sugar transporter sugar binding protein	5	1	K17329	dasA	map02010	ABC transporters
gene8427	sugar ABC transporter permease	1	0	K17330	dasB	map02010	ABC transporters
gene8428	Maltose/maltodextrin ABC transporter permease MalG	1	0	K17331	dasC	map02010	ABC transporters
gene8447	threonine dehydratase	0	0	K01754	ilvA	map00260;map00290	Glycine, serine and threonine metabolism;Valine, leucine and isoleucine biosynthesis
gene8465	alkaline phosphatase	1	0	K01077	phoA	map00730;map02020;map00790	Thiamine metabolism;Two-component system;Folate biosynthesis
gene8466	lipase class 2	7	0	K01046	lip	map00561	Glycerolipid metabolism

Supplementary Table 5 SM-BGCs predicted in the genome of *S. albulus* CICC11022.

SM-BGC number	Type of the secondary metabolite biosynthetic gene cluster	Location on genome		Most similar known cluster	Type of the secondary metabolite	Similarity
		From	To			
1	butyrolactone	180,712	191,566	chalcomycin A	Polyketide	7%
2	T1PKS,NRPS-like	213,859	262,246	ansamitocin P-3	Polyketide	9%
3	CDPS	293,620	314,444			
4	NRPS,T1PKS,NRPS-like	376,415	432,141	gougerotin	Other	46%
5	NAPAA	448,876	484,894	stenothricin	NRP:Cyclic depsipeptide	13%
6	NRPS-like	730,215	773,316	tetarimycin A/tetarimycin B	Polyketide:Type II polyketide	5%
7	lanthipeptide-class-i	801,485	826,917	himastatin	NRP	8%
8	T1PKS,NRPS	949,448	1,040,625	salinamide A/salinamide B/salinamide C/salinamide D/salinamide E/salinamide F/desmethylsalinamide C/desmethylsalinamide E	NRP:Cyclic depsipeptide+Polyketide:Modular type I polyketide	14%
9	NRPS,NRPS-like	1,247,916	1,298,625	glycinocin A	NRP	9%
10	T1PKS,NRPS-like,transAT-PKS-like	1,319,807	1,369,529	salinosporamide A	NRP+Polyketide	23%
11	T1PKS,NRPS-like	1,512,499	1,622,250	tetramycin B	Polyketide	100%
12	RiPP-like	1,677,818	1,688,105	conglobatin	NRP	15%
13	T2PKS	1,720,685	1,793,200	spore pigment	Polyketide	83%

14	NI-siderophore	2,590,828	2,620,630	legonoxamine A/desferrioxamine B/legonoxamine B	Other	100%
15	ectoine	2,686,650	2,697,060	ectoine	Other:Ectoine	100%
16	LAP	4,941,811	4,965,158	azolemycin B/azolemycin A/azolemycin D/azolemycin C	RiPP	22%
17	terpene	4,985,679	5,007,976	geosmin	Terpene	100%
18	NAPAA	5,665,974	5,700,188			
19	transAT-PKS,NRPS-like,NRPS	6,145,431	6,253,440	oxazolepoxidomycin A	NRP+Polyketide	86%
20	NI-siderophore	6,684,900	6,717,583	kinamycin	Polyketide	19%
21	T3PKS,butyrolactone,NRPS,PKS-like	6,776,394	6,891,284	griseoviridin/fijimycin A	NRP:Cyclic depsipeptide+Polyketide:Trans-AT type I polyketide	13%
22	RiPP-like	6,926,598	6,937,431			
23	lanthipeptide-class-i	7,280,459	7,305,460			
24	lanthipeptide-class-v,T2PKS,oligosaccharide	7,513,183	7,594,209	warkmycin CS1/warkmycin CS2	Polyketide+Saccharide:Hybrid/tailoring saccharide	97%
25	NRPS,nucleoside	7,596,386	7,639,070	toyocamycin	Other	100%
26	terpene	7,749,844	7,776,491	hopene	Terpene	61%

27	pyrrolidine,NAPAA,butyrolactone	8,020,564	8,070,981	anisomycin	Other	100%
28	butyrolactone	8,326,100	8,336,984	coelimycin P1	Polyketide:Modular type I polyketide	8%
29	CDPS	8,386,840	8,407,547			
30	NRPS-like,lanthipeptide-class-iii	8,524,123	8,567,092	SapB	RiPP:Lanthipeptide	100%
31	butyrolactone	8,609,171	8,620,106	lactonamycin	Polyketide	5%
32	NAPAA	8,856,616	8,891,872	stenothricin	NRP:Cyclic depsipeptide	13%
33	hydrogen-cyanide,RRE-containing,T1PKS,NAPAA,terpene	8,919,421	9,102,886	nystatin A1	Polyketide:Modular type I polyketide+Saccharide:Hybrid/tailoring saccharide	95%
34	NRPS-like,T1PKS	9,167,598	9,215,162	kedarcidin	NRP+Polyketide:Iterative type I polyketide+Polyketide:Enediyne type I polyketide	4%
35	CDPS	9,219,319	9,240,038	albonoursin	Other	50%
36	T1PKS	9,337,673	9,385,388	A54145	NRP	3%

Supplementary Table 6 Metabolites that were upregulated both intracellularly and extracellularly.

Number	Metabolite	CAS ID	KEGG Compound ID	M/Z	Retention time	Mode	Adducts	Formula
1	N-Acetylcadaverine	32343-73-0	-	145.1348	1.501	pos	M+H	C7H16N2O
2	6-Aminopenicillanic acid	551-16-6	C02954	217.0659	2.6823	pos	M+H-H_2O, M+H	C8H12N2O3S
3	Pantothenic acid	79-83-4	C00864	220.1197	2.961	pos	M+H, M+Na, M+H-H_2O, M+H-2H_2O	C9H17NO5
4	N6-Acetyl-L-lysine	692-04-6	C02727	171.1143	3.009	pos	M+H-H_2O	C8H16N2O3
5	2-(1-Naphthyl)acetamide	86-86-2	C18533	203.1195	5.5536	pos	M+NH_4	C12H11NO
6	3-Ureidopropionic acid	462-88-4	C02642	133.062	1.3019	pos	M+H-H_2O, M+H	C4H8N2O3
7	Hypoxanthine	68-94-0	C00262	137.047	1.6924	pos	M+H, M+Na	C5H4N4O
8	3-Oxododecanoic acid	-	C02367	467.2798	6.1296	pos	2M+K	C12H22O3
9	PC(PGJ2/2:0)	-	-	629.3249	3.5832	pos	2M+3H_2O+2H	C30H50NO10P
10	Prolyl-Asparagine	-	-	230.1155	2.7222	pos	M+H	C9H15N3O4
11	Beta-Alanyl-L-lysine	-	C05341	182.1304	2.5471	pos	M+H-2H_2O	C9H19N3O3
12	*p*-Coumaric Acid	4501-31-9	C02646	147.0453	2.0277	pos	M+H-H_2O	C9H8O3
13	Xanthosine	146-80-5	C01762	283.0682	3.3146	neg	M-H, M+Na-2H	C10H12N4O6
14	3-hydroxypentadecanoic acid	-	-	257.2119	6.5758	neg	M-H, M+FA-H, 2M-H	C15H30O3
15	Indolelactic acid	7417-65-4	C02043	204.0659	5.5422	neg	M-H	C11H11NO3
16	Salicyluric acid	487-54-7	C07588	194.0451	6.0119	neg	M-H	C9H9NO4
17	LysoPC(18:4(6Z,9Z,12Z,15Z))	-	-	560.3008	6.1762	neg	M+FA-H	C26H46NO7P
18	Ethyl 2-hydroxyisovalerate	-	-	145.0859	5.6284	neg	M-H	C7H14O3
19	Threoninyl-Isoleucine	129050-49-3	-	509.2871	6.0041	neg	M+FA-H, 2M+FA-H	C10H20N2O4
20	Xanthine	69-89-6	C00385	151.0251	1.9494	neg	M-H	C5H4N4O2
21	Gamma-D-Glutamylglycine	-	-	203.0666	0.964	neg	M-H	C7H12N2O5

Sequence of *SP43-SR41-gapN*

(5′ flank-*Xba* I-*SP43* promoter-*SR41* RBS-*gapN* gene-*BamH* I-3′ flank)

gcacgctctagatgttcacattcgaaccgtctcTGCTTTGACACGGACAAGCGCTATGGTGTAA
AGTCGTGGCCACACCTATCTAAGTAAGGAGTAGGCTGAATGACCAAGCAGT
ACAAGAACTACGTCAACGGCGAGTGGAAGCTGTCCGAGAACGAGATCAA
GATCTACGAGCCGGCCTCCGGCGCCGAGCTGGGCTCCGTCCCGGCCATGTC
CACCGAGGAGGTCGACTACGTCTACGCCTCCGCCAAGAAGGCCCAGCCGG
CCTGGCGCTCCCTGTCCTACATCGAGCGCGCCGCCTACCTGCACAAGGTCG
CCGACATCCTGATGCGCGACAAGGAGAAGATCGGCGCCGTCCTGTCCAAG
GAGGTCGCCAAGGGCTACAAGTCCGCCGTCTCCGAGGTCGTCCGCACCGC
CGAGATCATCAACTACGCCGCCGAGGAGGGCCTGCGCATGGAGGGCGAGG
TCCTGGAGGGCGGCTCCTTCGAGGCCGCCTCCAAGAAGAAGATCGCCGTC
GTCCGCCGCGAGCCGGTCGGCCTGGTCCTGGCCATCTCCCCGTTCAACTAC
CCGGTCAACCTGGCCGGCTCCAAGATCGCCCCGGCCCTGATCGCCGGCAA
CGTCATCGCCTTCAAGCCGCCGACCCAGGGCTCCATCTCCGGCCTGCTGCT
GGCCGAGGCCTTCGCCGAGGCCGGCCTGCCGGCCGGCGTCTTCAACACCA
TCACCGGCCGCGGCTCCGAGATCGGCGACTACATCGTCGAGCACCAGGCC
GTCAACTTCATCAACTTCACCGGCTCCACCGGCATCGGCGAGCGCATCGGC
AAGATGGCCGGCATGCGCCCGATCATGCTGGAGCTGGGCGGCAAGGACTC
CGCCATCGTCCTGGAGGACGCCGACCTGGAGCTGACCGCCAAGAACATCA
TCGCCGGCGCCTTCGGCTACTCCGGCCAGCGCTGCACCGCCGTCAAGCGC
GTCCTGGTCATGGAGTCCGTCGCCGACGAGCTGGTCGAGAAGATCCGCGA
GAAGGTCCTGGCCCTGACCATCGGCAACCCGGAGGACGACGCCGACATCA
CCCCGCTGATCGACACCAAGTCCGCCGACTACGTCGAGGGCCTGATCAAC
GACGCCAACGACAAGGGCGCCACCGCCCTGACCGAGATCAAGCGCGAGG
GCAACCTGATCTGCCCGATCCTGTTCGACAAGGTCACCACCGACATGCGCC
TGGCCTGGGAGGAGCCGTTCGGCCCGGTCCTGCCGATCATCCGCGTCACCT
CCGTCGAGGAGGCCATCGAGATCTCCAACAAGTCCGAGTACGGCCTGCAG
GCCTCCATCTTCACCAACGACTTCCCGCGCGCCTTCGGCATCGCCGAGCAG
CTGGAGGTCGGCACCGTCCACATCAACAACAAGACCCAGCGCGGCACCGA
CAACTTCCCGTTCCTGGGCGCCAAGAAGTCCGGCGCCGGCATCCAGGGCG
TCAAGTACTCCATCGAGGCCATGACCACCGTCAAGTCCGTCGTCTTCGACA
TCAAGTAAggatccgtcgtc

Supplementary Figure 1 Description and assembled sequences used in this study.

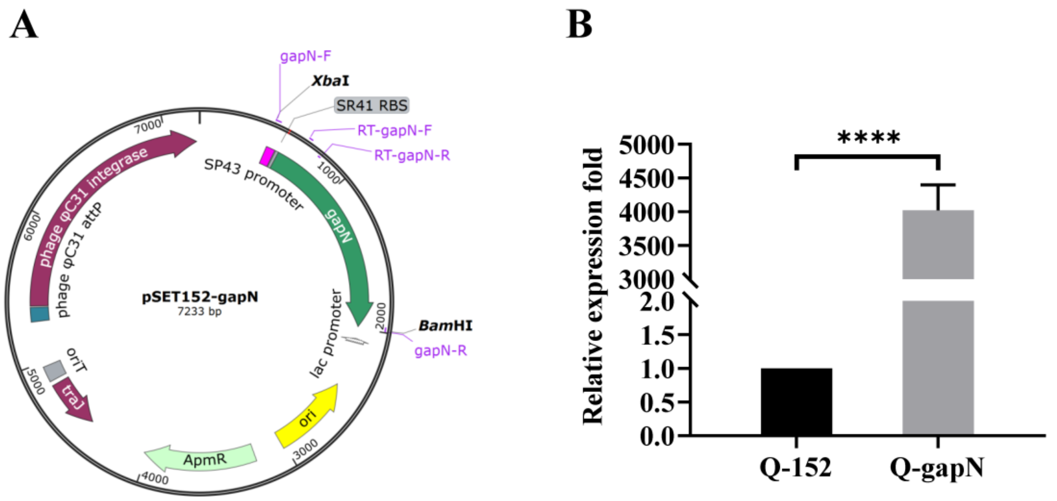

Supplementary Figure 2 Construction and verification of the *gapN* heterologously expressing strain *S. albulans* Q-gapN. (A) Diagram of the *gapN* gene expression vector of pSET152-gapN. (B) Relative expression fold of *gapN* gene between Q-gapN and Q-152. ****, $P < 0.0001$.

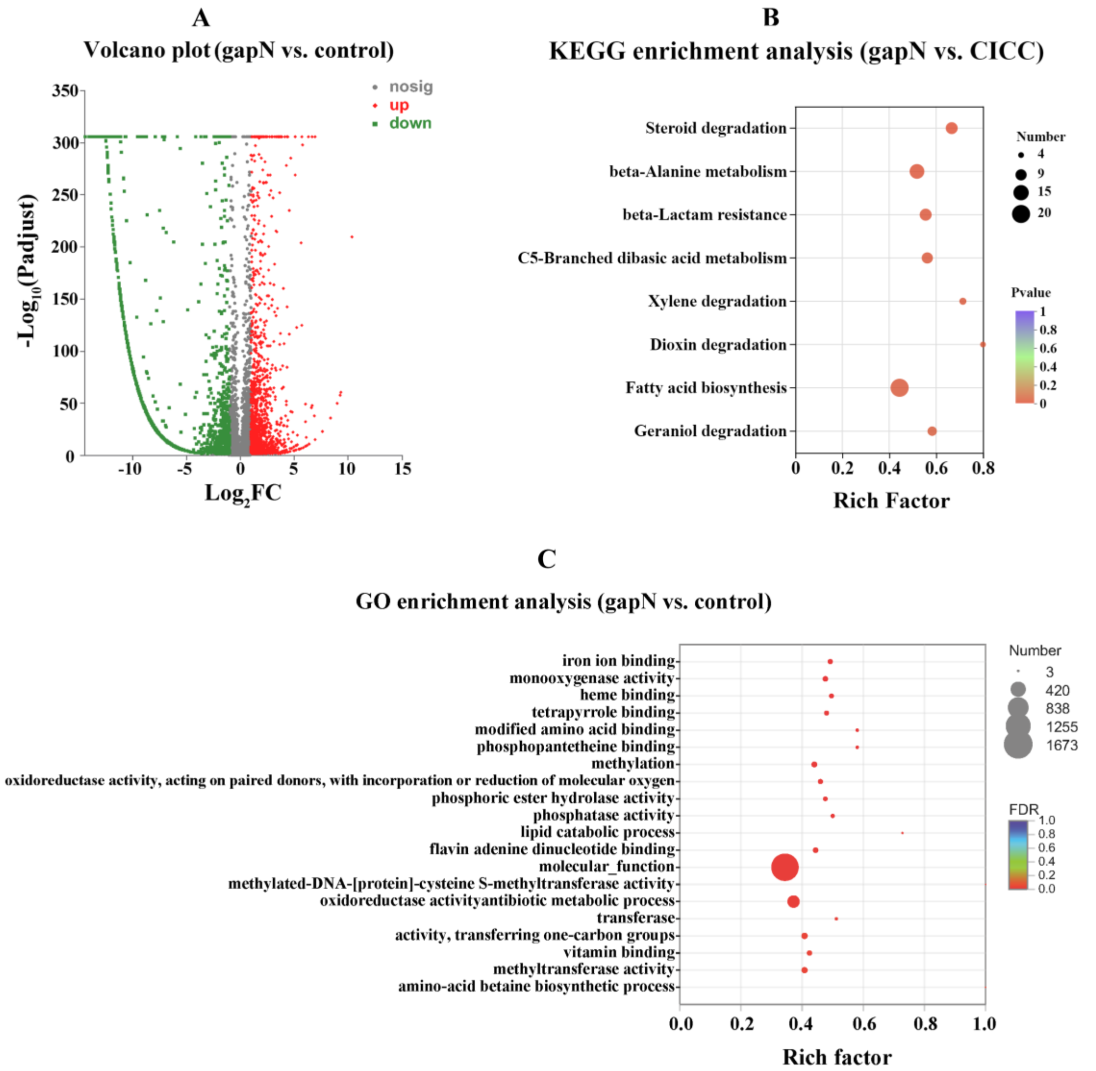

Supplementary Figure 3 Transcriptomic comparison of the *gapN* expressing strain (gapN) and the control strain (control). (A) Volcanic map of the significant DEGs between gapN and control. (B) KEGG enrichment analyses between gapN and control. (C) GO enrichment analyses between gapN and control.

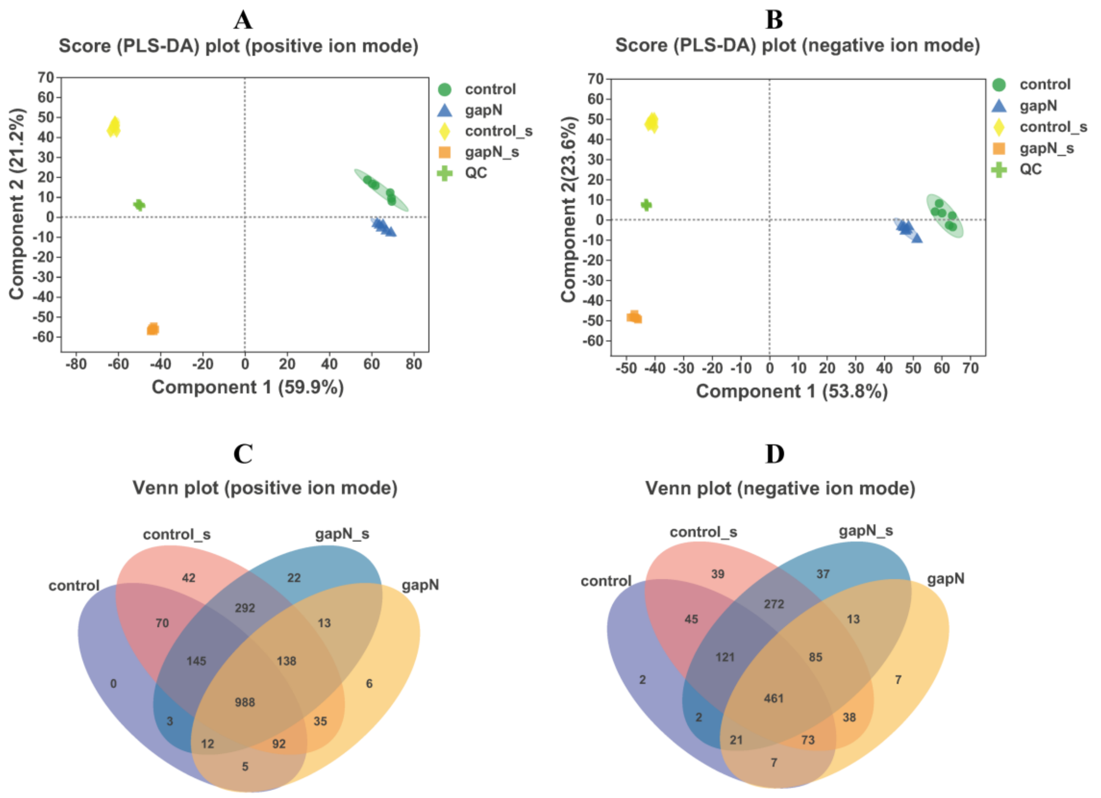

Supplementary Figure 4 PLS-DA plot and Venn plot analyses of the metabolomic results. (A) PLS-DA plot analysis of the four groups in the positive ion mode. (B) PLS-DA plot analysis of the four groups in the negative ion mode. (C) Venn plot analysis of the four groups in the positive ion mode. (D) Venn plot analysis of the four groups in the negative ion mode.

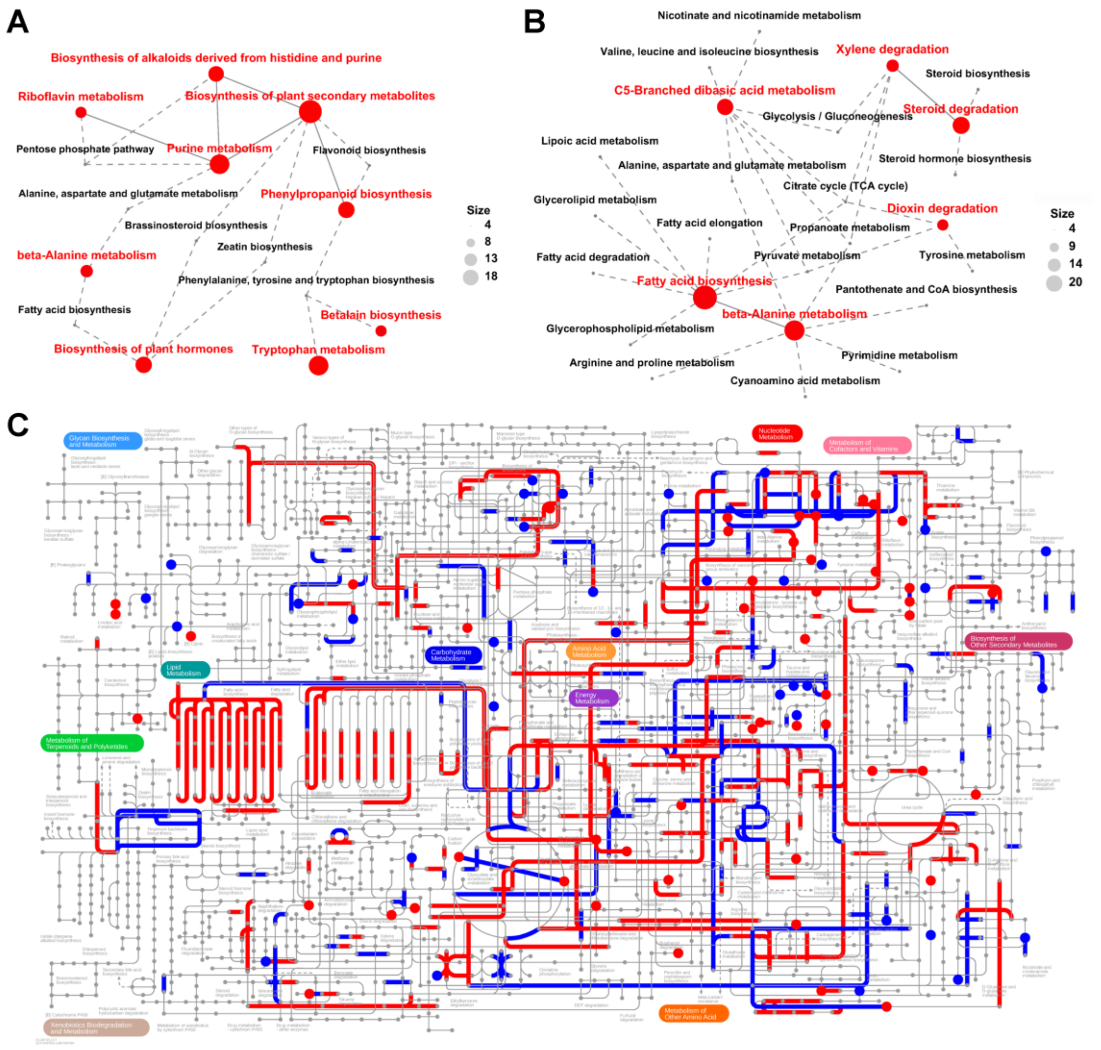

Supplementary Figure 5 Combination of transcriptome and metabolomic analyses. (A) Network diagram of the KEGG pathway enrichment analysis of the differential intracellular metabolites. (B) Network diagram of the KEGG pathway enrichment analysis of the differentially expressed genes. The red nodes in Figs. S5A and 5B represent the enriched KEGG pathways ($P < 0.05$). Node size represents the number of genes enriched in each pathway. (C) iPath analysis of the differentially expressed genes and intracellular metabolites. The red and blue lines represent the genes that were significantly up- and downregulated, respectively. The red and blue nodes represent the metabolites that were significantly up- and downregulated, respectively.

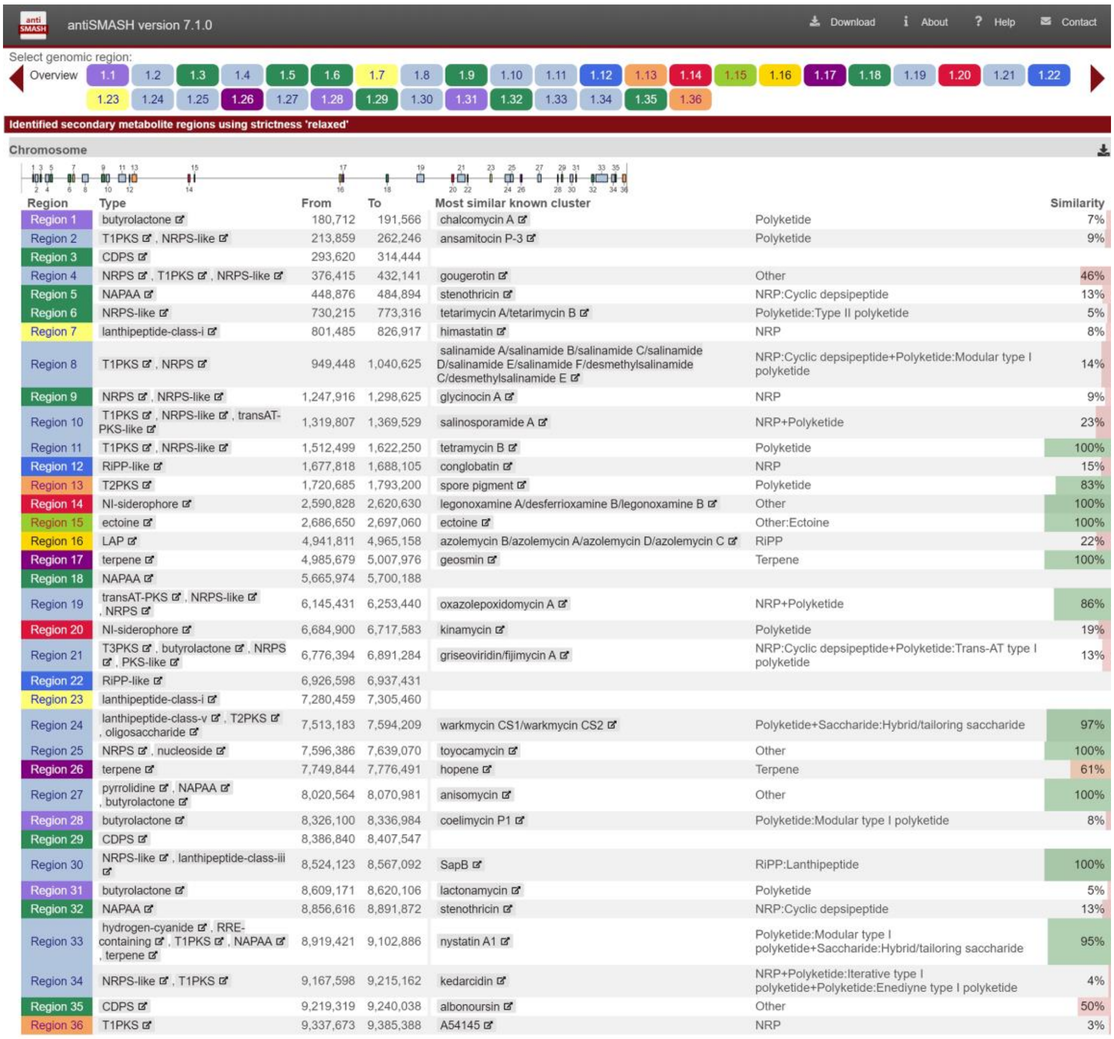

Region	Type	From	To	Most similar known cluster		Similarity
Region 1	butyrolactone	180,712	191,566	chalcomycin A	Polyketide	7%
Region 2	T1PKS , NRPS-like	213,859	262,246	ansamitocin P-3	Polyketide	9%
Region 3	CDPS	293,620	314,444			
Region 4	NRPS , T1PKS , NRPS-like	376,415	432,141	gougerotin	Other	46%
Region 5	NAPAA	448,876	484,894	stenothricin	NRP:Cyclic depsipeptide	13%
Region 6	NRPS-like	730,215	773,316	tetarimycin A/tetarimycin B	Polyketide:Type II polyketide	5%
Region 7	lanthipeptide-class-i	801,485	826,917	himastatin	NRP	8%
Region 8	T1PKS , NRPS	949,448	1,040,625	salinamide A/salinamide B/salinamide C/salinamide D/salinamide E/salinamide F/desmethylsalinamide C/desmethylsalinamide E	NRP:Cyclic depsipeptide+Polyketide:Modular type I polyketide	14%
Region 9	NRPS , NRPS-like	1,247,916	1,298,625	glycinocin A	NRP	9%
Region 10	T1PKS , NRPS-like , transAT-PKS-like	1,319,807	1,369,529	salinosporamide A	NRP+Polyketide	23%
Region 11	T1PKS , NRPS-like	1,512,499	1,622,250	tetramycin B	Polyketide	100%
Region 12	RiPP-like	1,677,818	1,688,105	conglobatin	NRP	15%
Region 13	T2PKS	1,720,685	1,793,200	spore pigment	Polyketide	83%
Region 14	NI-siderophore	2,590,828	2,620,630	legonoxamine A/desferrioxamine B/legonoxamine B	Other	100%
Region 15	ectoine	2,686,650	2,697,060	ectoine	Other:Ectoine	100%
Region 16	LAP	4,941,811	4,965,158	azolemycin B/azolemycin A/azolemycin D/azolemycin C	RiPP	22%
Region 17	terpene	4,985,679	5,007,976	geosmin	Terpene	100%
Region 18	NAPAA	5,665,974	5,700,188			
Region 19	transAT-PKS , NRPS-like , NRPS	6,145,431	6,253,440	oxazolepoxidomycin A	NRP+Polyketide	86%
Region 20	NI-siderophore	6,684,900	6,717,583	kinamycin	Polyketide	19%
Region 21	T3PKS , butyrolactone , NRPS , PKS-like	6,776,394	6,891,284	griseoviridin/fijimycin A	NRP:Cyclic depsipeptide+Polyketide:Trans-AT type I polyketide	13%
Region 22	RiPP-like	6,926,598	6,937,431			
Region 23	lanthipeptide-class-i	7,280,459	7,305,460			
Region 24	lanthipeptide-class-v , T2PKS , oligosaccharide	7,513,183	7,594,209	warkmycin CS1/warkmycin CS2	Polyketide+Saccharide:Hybrid/tailoring saccharide	97%
Region 25	NRPS , nucleoside	7,596,386	7,639,070	toyocamycin	Other	100%
Region 26	terpene	7,749,844	7,776,491	hopene	Terpene	61%
Region 27	pyrrolidine , NAPAA , butyrolactone	8,020,564	8,070,981	anisomycin	Other	100%
Region 28	butyrolactone	8,326,100	8,336,984	coelimycin P1	Polyketide:Modular type I polyketide	8%
Region 29	CDPS	8,386,840	8,407,547			
Region 30	NRPS-like , lanthipeptide-class-iii	8,524,123	8,567,092	SapB	RiPP:Lanthipeptide	100%
Region 31	butyrolactone	8,609,171	8,620,106	lactonamycin	Polyketide	5%
Region 32	NAPAA	8,856,616	8,891,872	stenothricin	NRP:Cyclic depsipeptide	13%
Region 33	hydrogen-cyanide , RRE-containing , T1PKS , NAPAA , terpene	8,919,421	9,102,886	nystatin A1	Polyketide:Modular type I polyketide+Saccharide:Hybrid/tailoring saccharide	95%
Region 34	NRPS-like , T1PKS	9,167,598	9,215,162	kedarcidin	NRP+Polyketide:Iterative type I polyketide+Polyketide:Enediyne type I polyketide	4%
Region 35	CDPS	9,219,319	9,240,038	albonoursin	Other	50%
Region 36	T1PKS	9,337,673	9,385,388	A54145	NRP	3%

Supplementary Figure 6 SM-BGCs predicted by antiSMASH in the genome of *S. albulus* CICC11022.

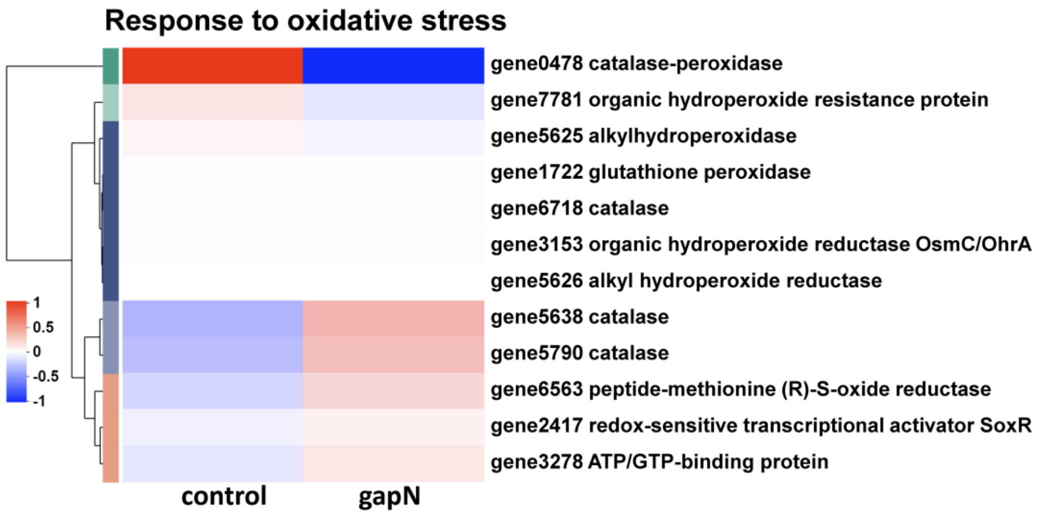

Supplementary Figure 7 Heatmap of genes involved in the “response to oxidative stress” pathway
